# Maternal-Fetal Immune Responses in Pregnant Women Infected with SARS-CoV-2

**DOI:** 10.21203/rs.3.rs-362886/v1

**Published:** 2021-03-31

**Authors:** Valeria Garcia-Flores, Roberto Romero, Yi Xu, Kevin Theis, Marcia Arenas-Hernandez, Derek Miller, Azam Peyvandipour, Jose Galaz, Dustyn Levenson, Gaurav Bhatti, Meyer Gershater, Errile Pusod, David Kracht, Violetta Florova, Yaozhu Leng, Li Tao, Megan Faucett, Robert Para, Chaur-Dong Hsu, Gary Zhang, Adi L. Tarca, Roger Pique-Regi, Nardhy Gomez-Lopez

**Affiliations:** 1 Perinatology Research Branch, Division of Obstetrics and Maternal-Fetal Medicine, Division of Intramural Research, *Eunice Kennedy Shriver* National Institute of Child Health and Human Development, National Institutes of Health, U.S. Department of Health and Human Services (NICHD/NIH/DHHS); Bethesda, Maryland, 20892 and Detroit, Michigan, 48201, USA; 2 Department of Obstetrics and Gynecology, Wayne State University School of Medicine, Detroit, Michigan, 48201, USA; 3 Department of Obstetrics and Gynecology, University of Michigan, Ann Arbor, Michigan, 48109, USA; 4 Department of Epidemiology and Biostatistics, Michigan State University, East Lansing, Michigan, 48824, USA; 5 Center for Molecular Medicine and Genetics, Wayne State University, Detroit, Michigan, 48201, USA; 6 Detroit Medical Center, Detroit, Michigan, 48201, USA; 7 Department of Obstetrics and Gynecology, Florida International University, Miami, Florida, 22 33199, USA; 8 Department of Biochemistry, Microbiology and Immunology, Wayne State University School of Medicine, Detroit, Michigan, 48201, USA; 9 Department of Physiology, Wayne State University School of Medicine, Detroit, Michigan 48201, USA;; 10 Department of Computer Science, Wayne State University College of Engineering, Detroit, Michigan, 48201, USA; 11 Lead contact

**Keywords:** Cytokines, COVID-19, Fetus, Immunoglobulins, IgG, IgM, Macrophages, Neonatal Immunity, Placenta, T cells, Umbilical Cord

## Abstract

Pregnant women are a high-risk population for severe/critical COVID-19 and mortality. However, the maternal-fetal immune responses initiated by SARS-CoV-2 infection, and whether this virus is detectable in the placenta, are still under investigation. Herein, we report that SARS-CoV-2 infection during pregnancy primarily induced specific maternal inflammatory responses in the circulation and at the maternal-fetal interface, the latter being governed by T cells and macrophages. SARS-CoV-2 infection during pregnancy was also associated with a cytokine response in the fetal circulation (i.e. umbilical cord blood) without compromising the cellular immune repertoire. Moreover, SARS-CoV-2 infection neither altered fetal cellular immune responses in the placenta nor induced elevated cord blood levels of IgM. Importantly, SARS-CoV-2 was not detected in the placental tissues, nor was the sterility of the placenta compromised by maternal viral infection. This study provides insight into the maternal-fetal immune responses triggered by SARS-CoV-2 and further emphasizes the rarity of placental infection.

## INTRODUCTION

To date, over 65,000 pregnant women in the United States have been infected with SARS-CoV-2^1^, the virus responsible for the coronavirus disease 2019 (COVID-19). During pregnancy, SARS-CoV-2 infection can lead to variable outcomes, which range from experiencing no symptoms to developing severe/critical disease^2,3^. Most pregnant women with SARS-CoV-2 infection are asymptomatic or only experience mild symptoms^4,5^. Regardless, in the first six months of the COVID-19 pandemic, it was documented that pregnant women with SARS-CoV-2 were at an increased risk for hospitalization, mechanical ventilation, intensive care unit admission, and preterm birth^2,3,6–8^, but rates of maternal mortality were reported to be similar between pregnant and non-pregnant women^6^. More recently, it has been clearly shown that pregnant women are at a high risk for severe/critical disease and mortality as well as preterm birth^9–12^. Therefore, investigating host immune responses in pregnant women infected with SARS-CoV-2, even if they are asymptomatic, is timely.

Most neonates born to infected women test negative for SARS-CoV-2, and the majority of those testing positive for the virus present symptoms that are not severe^8,13^. For the latter group, the timing of mother-to-child transmission (i.e. vertical transmission) of SARS-CoV-2 is still unclear, since this can occur *in utero*, intrapartum, or early in the postnatal period^14^. Yet, while rare^14^, there is already evidence of SARS-CoV-2 *in utero* vertical transmission^15,16^, which is likely to occur through the hematogenous route (i.e. bloodstream infection)^17^. In such cases, the virus must cross the maternal-fetal interface by infecting the syncytiotrophoblast layer of the placenta to gain access to the fetal circulation. The mechanisms whereby SARS-CoV-2 infects placental cells are still under investigation; however, it is well accepted that coronaviruses can enter host cells via two main canonical mechanisms^18,19^: 1) the direct pathway, in which host cells are required to express both the angiotensin-converting enzyme 2 (ACE-2) receptor^20^ and the serine protease TMPRSS2^21^; and 2) the endosomal route, in which cell entry can be mediated by ACE-2 alone. Using both single-cell and single-nuclear RNA sequencing, we have previously shown that the co-expression of ACE-2 and TMPRSS2 is negligible in first, second, and third trimester placental cells^22^. Subsequent investigations demonstrated that the ACE-2 protein was polarized to the stromal (fetal) side of the syncytiotrophoblast and TMPRSS2 limited to the villous endothelium^23,24^. Yet, placental cells can express non-canonical cell entry mediators such as cathepsin L (CSTL), FURIN, and SIGLEC1, among others^22^. Furthermore, SARS-CoV-2 infection can be associated with vascular damage in pregnant women, in whom ischemic injury of the placenta may facilitate viral cell entry^25^. Therefore, SARS-CoV-2 can infect placental cells, as has already been reported^26–28^; however, placental infection alone is not considered confirmatory evidence of *in utero* vertical transmission^14^. Nonetheless, it is possible that the maternal inflammatory response induced by SARS-CoV-2 infection has deleterious effects on the offspring. Therefore, investigating the host immune response in the umbilical cord blood as well as at the site of maternal-fetal interactions (i.e. the maternal-fetal interface) may shed light on the adverse effects of SARS-CoV-2 infection during pregnancy.

In the current study, we undertook a multidisciplinary approach that included the detection of SARS-CoV-2 IgM/IgG, multiplex cytokine assays, immunophenotyping, single-cell transcriptomics, and viral RNA and protein detection, together with the assessment of the microbiome diversity and histopathology of the placenta, to characterize the maternal-fetal immune responses triggered by SARS-CoV-2 during pregnancy.

## RESULTS

### Characteristics of the study population

A total of 15 pregnant women were enrolled in our study. The demographic and clinical characteristics of the study population are displayed in Supplementary Table 1. Maternal blood samples were collected upon admission, prior to administration of any medication. Seven pregnant women tested RT-PCR positive (nasopharyngeal swab) for SARS-CoV-2; five were asymptomatic, one had mild symptoms (e.g. fever, tachycardia), and one was diagnosed as having severe COVID-19 (requiring oxygen supplementation). SARS-CoV-2 positive and control non-infected women all delivered term neonates. Neonates were not RT-qPCR tested for SARS-CoV-2; thus, infection status throughout the manuscript refers solely to the mother. No differences in demographic and clinical characteristics were found between the study groups, including Apgar scores and placental histopathological lesions.

### Pregnant women with SARS-CoV-2 infection and their neonates exhibit distinct IgM responses

**Figure 1.**
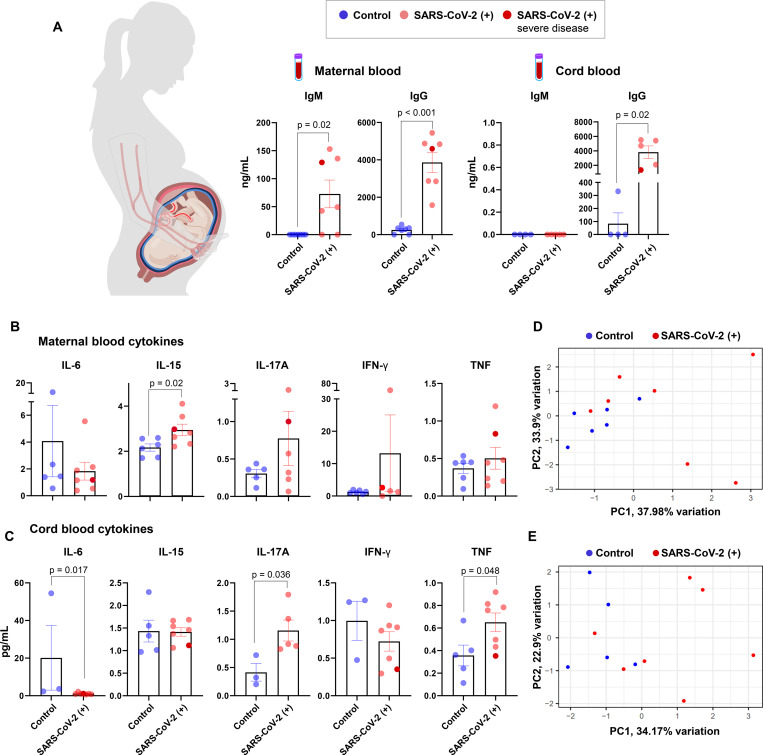
Serological and cytokine responses in pregnant women with SARS-CoV-2 infection and their neonates. **(A)** Serum concentrations of IgM and IgG in the maternal peripheral blood (n = 7 per group) (left panel) and cord blood (n = 4–5 per group) (right panel) from SARS-CoV-2 (+) or healthy pregnant women. Bar plots represent mean and standard error of the mean. Differences between two groups were evaluated by Mann-Whitney U tests. **(B)** Plasma concentrations of IL-6, IL-15, IL-17A, IFN-γ, and TNF in the maternal peripheral blood (n = 6–7 per group). Blue dots indicate healthy pregnant women, light red dots indicate SARS-CoV-2 (+) pregnant women, and the dark red dot indicates one patient with severe COVID-19 disease. **(C)** Plasma concentrations of IL-6, IL-15, IL-17A, IFN-γ, and TNF in the cord blood (n = 5–7 per group). Blue dots indicate healthy pregnant women, light red dots indicate SARS-CoV-2 (+) pregnant women, and the dark red dot indicates one patient with severe COVID-19 disease. **(D)** Scatter plot of the first two principal components (PC1 and PC2) from cytokine concentrations in the maternal plasma. Blue dots indicate healthy pregnant women and red dots indicate SARS-CoV-2 (+) pregnant women. **(E)** Scatter plot of PC1 and PC2 from cytokine concentrations in the cord blood plasma. Blue dots indicate healthy pregnant women and red dots indicate SARS-CoV-2 (+) pregnant women. Bar plots represent mean and standard error of the mean. Differences in cytokine concentrations between groups were evaluated by Mann-Whitney U-tests. Differences in PC1 values between SARS-CoV-2 (+) and healthy pregnant women were assessed using two-sample student’s t-tests. P values are considered significant when p < 0.05.

Previous studies have shown that maternal IgG antibodies are transferred across the placenta in both symptomatic and asymptomatic women infected with SARS-CoV-2^29^. In addition, there is evidence showing that neonates born to mothers with COVID-19 can have detectable SARS-CoV-2 IgM as well as IgG^16,30^. The presence of IgG is likely due to the passive transfer of this immunoglobulin from the mother to the fetus across the placenta. However, detectable levels of IgM suggest that the fetus was infected with SARS-CoV-2, given that this immunoglobulin cannot cross the placenta due to its large molecular weight. Therefore, we first determined the concentrations of SARS-CoV-2-specific IgM and IgG in the maternal and umbilical cord blood (hereafter referred to as ‘cord blood’). As expected, pregnant women with SARS-CoV-2 infection had higher levels of IgM and IgG than controls (Fig. 1A). The IgM and IgG serum levels of the pregnant woman with severe COVID-19 were similar to those without symptoms or with mild symptoms. In addition, IgG was increased in the cord blood of neonates born to women infected with SARS-CoV-2 infection but IgM was undetected, similar to control neonates (Fig. 1A). Therefore, serological data imply that in our study population, which is largely asymptomatic for COVID-19, none of the neonates seems to be infected with SARS-CoV-2.

### Pro-inflammatory cytokine responses are displayed in the circulation of pregnant women with SARS-CoV-2 infection and their neonates

The pathophysiology of SARS-CoV-2 infection includes a cytokine storm in the systemic circulation, which can lead to multi-organ damage^31,32^. Hence, we next determined the systemic cytokine response in mothers and neonates by measuring the concentrations of 20 cytokines in maternal and cord blood plasma. Pregnant women infected with SARS-CoV-2 had increased systemic concentrations of IL-15 (0.43-log2 fold change) and tended to have higher concentrations of IFN-γ (1.84-log2 fold change) and IL-8 (1.25-log2 fold change) compared to control mothers; yet, these increments did not reach statistical significance (Fig. 1B, Supplementary Fig. 1, Supplementary Table 2). Such changes were not driven by the severe COVID-19 case. Neonates born to women infected with SARS-CoV-2 had increased concentrations of IL-17A (1.61-log2 fold change) and TNF (1.01-log2 fold change), but lower concentrations of IL-6 (−2.90-log2 fold change), compared to those born to control mothers (Fig. 1C). In addition, neonates born to women who tested positive for SARS-CoV-2 tended to display higher concentrations of several cytokines including IL-12/IL-23p40 (1.32-log2 fold change), VEGF (1.56-log2 fold change), IL-5 (1.23-log2 fold change), and IL-8 (0.99-log2 fold change) than those born to control mothers (Fig. 1C, Supplementary Fig. 2, Supplementary Table 2). Such inflammatory changes in the neonates were not solely driven by the severe COVID-19 case. Based on an unsupervised analysis, the primary source of variability in the maternal and fetal cytokine responses was the SARS-CoV-2 infection status (first principal components in Fig. 1D&E significant between groups, p<0.05 for both). These results show that a cytokine response is observed in both the maternal and fetal circulation upon maternal infection with SARS-CoV-2.

### Pregnant women with SARS-CoV-2 infection, but not their neonates, undergo a T-cell reduction in the circulation

**Figure 2.**
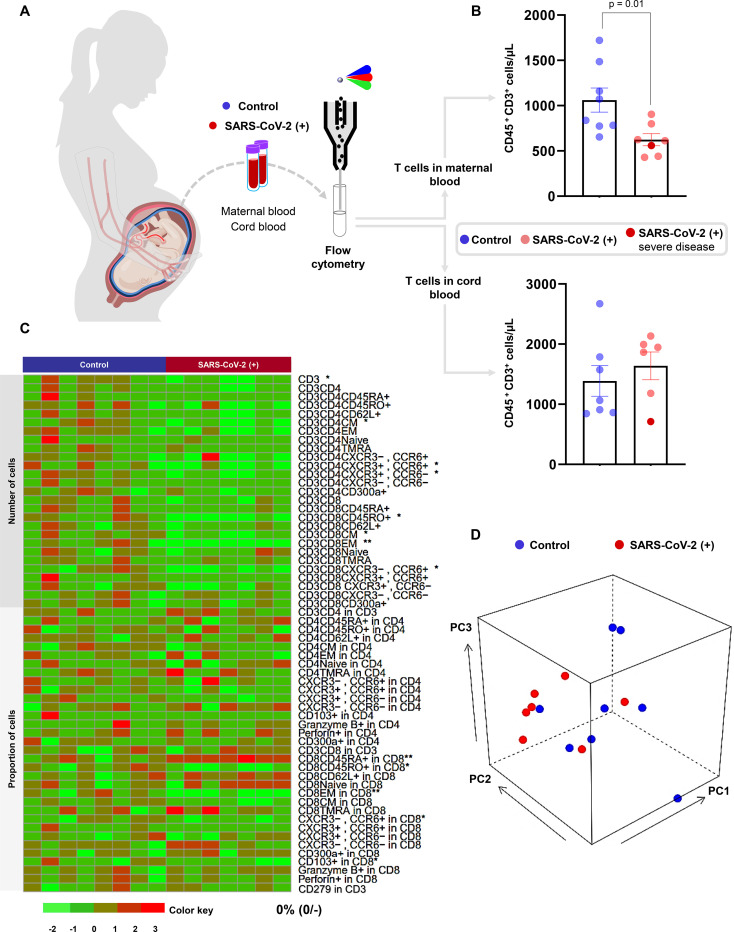
Immunophenotyping of T cells in pregnant women with SARS-CoV-2 infection and their neonates. **(A)** Maternal peripheral blood and cord blood were collected for immunophenotyping by flow cytometry. **(B)** Numbers of T cells in the maternal blood (n = 7–8 per group) and cord blood (n = 6–7 per group) from SARS-CoV-2 (+) or healthy pregnant women. Bar plots represent mean and standard error of the mean. Differences between groups were evaluated by Mann-Whitney U-tests. P values < 0.05 were used to denote a significant result. Blue dots indicate healthy pregnant women, light red dots indicate SARS-CoV-2 (+) pregnant women, and the dark red dot indicates one patient with severe COVID-19 disease. **(C)** Heatmap showing abundance (z-scores) for T cell subsets in the maternal blood from SARS-CoV-2 (+) or healthy pregnant women (n = 7–8 per group). Cell numbers and proportions are shown. Differences between groups were assessed using two-sample t-tests. P values were adjusted for multiple comparisons using false discovery rate (FDR) method to obtain q values. * q < 0.1; ** q < 0.05. Red and green indicate increased and decreased abundance, respectively. **(D)** Three-dimensional scatter plot showing the distribution of flow cytometry data from the maternal blood of SARS-CoV-2 (+) (red dots) or healthy pregnant women (blue dots) (n = 7–8 per group) based on principal component (PC)1, PC2, and PC3.

**Figure 3.**
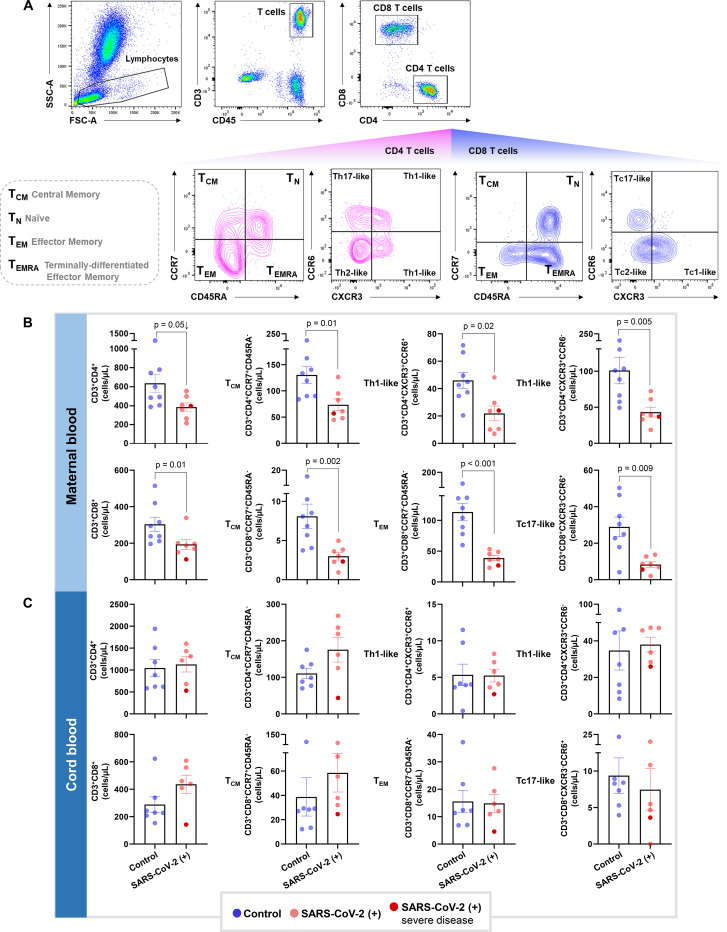
T cell subsets in pregnant women with SARS-CoV-2 infection and their neonates. **(A)** Representative gating strategy used to identify CD4^+^ and CD8^+^ T cells, and their respective subsets, within the total T cell population (CD45^+^CD3^+^ cells) in the maternal blood and cord blood. **(B)** Numbers of CD4^+^ T cells, CD4^+^ T_CM_, CXCR3^+^CCR6^+^ Th1-like cells, and CXCR3^+^CCR6^−^ Th1-like cells (upper row); and the numbers of CD8^+^ T cells, CD8^+^ T_CM_, CD8^+^ T_EM_, and Tc17-like cells (lower row) in the maternal blood from SARS-CoV-2 (+) or healthy pregnant women (n = 7–8). **(C)** Numbers of CD4^+^ T cells, CD4^+^ T_CM_, CXCR3^+^CCR6^+^ Th1-like cells, and CXCR3^+^CCR6^−^ Th1-like cells (upper row); and the numbers of CD8^+^ T cells, CD8^+^ T_CM_, CD8^+^ T_EM_, and Tc17-like cells (lower row) in the cord blood from SARS-CoV-2 (+) or healthy pregnant women (n = 6–7 per group). Bar plots represent mean and standard error of the mean. Differences between groups were evaluated by Mann-Whitney U-tests, with p < 0.05 being considered significant. Blue dots indicate healthy pregnant women, light red dots indicate SARS-CoV-2 (+) women, and the dark red dot indicates one patient with severe COVID-19 disease.

Previous studies have shown that patients with moderate or severe COVID-19 display alterations in their cellular immune responses in the peripheral circulation^32–34^. Therefore, we investigated whether pregnant women with SARS-CoV-2 infection and their neonates had changes in their cellular immune repertoire using immunophenotyping (Fig. 2A, Supplementary Fig. 3A). Immunophenotyping included the identification of general leukocyte subpopulations as well as monocyte, neutrophil, B-cell, and T-cell subsets. Neutrophil and monocyte function has also been implicated in the pathogenesis of SARS-CoV-2 infection^34–36^; therefore, reactive oxygen species (ROS) production by neutrophils and monocytes was also determined in maternal and cord blood (Supplementary Fig. 4A). No statistical differences were observed in the total number of general leukocyte subpopulations or in the monocyte, neutrophil, activated T-cell, and B-cell subsets (Supplementary Fig. 3B–F). Although neutrophils and monocytes produced ROS when stimulated, no differences were found between SARS-CoV-2 cases and controls in the maternal blood or in the cord blood (Supplementary Fig. 4B&C). Nonetheless, pregnant women with SARS-CoV-2 infection had reduced T-cell numbers, but their neonates did not display such a decline (Fig. 2B). Heatmap and principal component analysis (PCA) representations of the immunophenotyping of the maternal blood showed that SARS-CoV-2 infection mildly altered T-cell subsets (Fig. 2C&D). Specifically, pregnant women infected with SARS-CoV-2 had reduced numbers of CD4^+^ T cells, including T_CM_ and Th1-like cells, as well as CD8^+^ T cells, including T_CM_, T_EM_, and Tc17-like cells (Fig. 3A&B). Such changes were not solely driven by the severe COVID-19 case. Neonates born to women with SARS-CoV-2 infection did not display changes in the T-cell subsets that were affected in mothers (Fig. 3C). These data showed that pregnant women infected with SARS-CoV-2 undergo a reduction in T-cell subsets, including pro-inflammatory Th1- and Tc17-like cells, which is not translated to the neonatal T-cell repertoire.

### Single-cell RNA sequencing reveals perturbed maternal T-cell and macrophage responses at the maternal-fetal interface of women with SARS-CoV-2 infection

**Figure 4.**
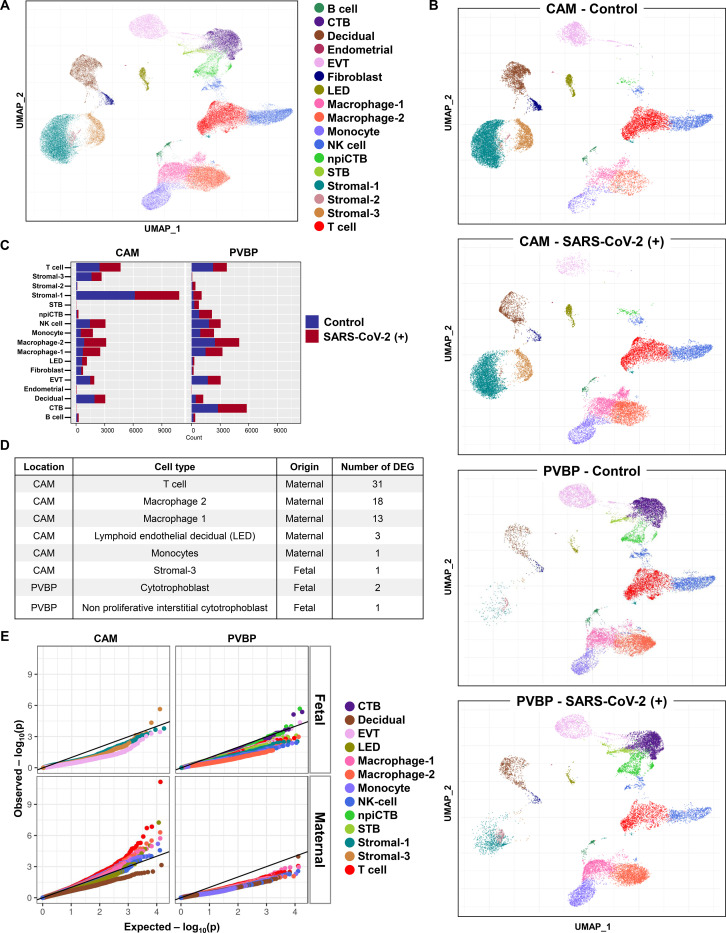
Single-cell RNA sequencing of the placental tissues of women with SARS-CoV-2 infection. **(A)** Uniform Manifold Approximation Plot (UMAP) showing the combined cell type classifications from the chorioamniotic membranes (CAM) and placental villi and basal plate (PVBP) of SARS-CoV-2 (+) or healthy pregnant women (n = 7–8 per group), where each dot represents a single cell. Abbreviations used are: CTB, cytotrophoblast; EVT, extravillous trophoblast; LED, lymphoid endothelial decidual cell; npiCTB, non-proliferative interstitial cytotrophoblast; STB, syncytiotrophoblast. **(B)** UMAP showing cell populations separated based on placental compartment (CAM and PVBP) from SARS-CoV-2 (+) or healthy pregnant women. **(C)** Bar plot showing the numbers of cells of each type in the CAM and PVBP of SARS-CoV-2 (+) or healthy pregnant women. **(D)** Number of differentially expressed genes (DEG) in each cell type from the CAM and PVBP with false discovery rate (FDR) adjusted p < 0.1. **(E)** Quantile-quantile (Q-Q) plot showing differential expression of all tested genes in each cell type of maternal or fetal origin from the CAM and PVBP samples. Deviation above the 1:1 line (solid black line) indicates enrichment.

Next, we investigated whether SARS-CoV-2 infection in the mother could alter cellular immune responses in the placenta, the organ that serves as the lungs, gut, kidneys, and liver of the fetus^37,38^. We performed single-cell RNA sequencing (scRNAseq) of the placental tissues including the basal plate (placental villous and basal plate, PVBP) and the chorioamniotic membranes (CAM) from pregnant women with SARS-CoV-2 infection and controls, using established methods. Consistent with our previous studies^22,39^, multiple cell clusters were identified in the placental tissues including lymphoid and myeloid immune cells, trophoblast cell types, stromal cells, and endometrial/decidual cells as well as endothelial cells (Fig. 4A). Differences in abundance among cell type clusters were observed between placental compartments as well as between tissues from women with SARS-CoV-2 infection and those from controls (Fig. 4B&C). Further analysis revealed that the majority of the differentially expressed genes (DEGs, Supplementary Table 3) between SARS-CoV-2 positive cases and controls belong to immune cells from the CAM, namely maternal T cells and macrophages (Fig. 4D&E). Lymphatic endothelial decidual (LED) cells of maternal origin displayed three DEGs between SARS-CoV-2 cases and controls. In general, fetal cell types were minimally altered by the presence of SARS-CoV-2 infection in the mother (Fig. 4D&E).

**Figure 5.**
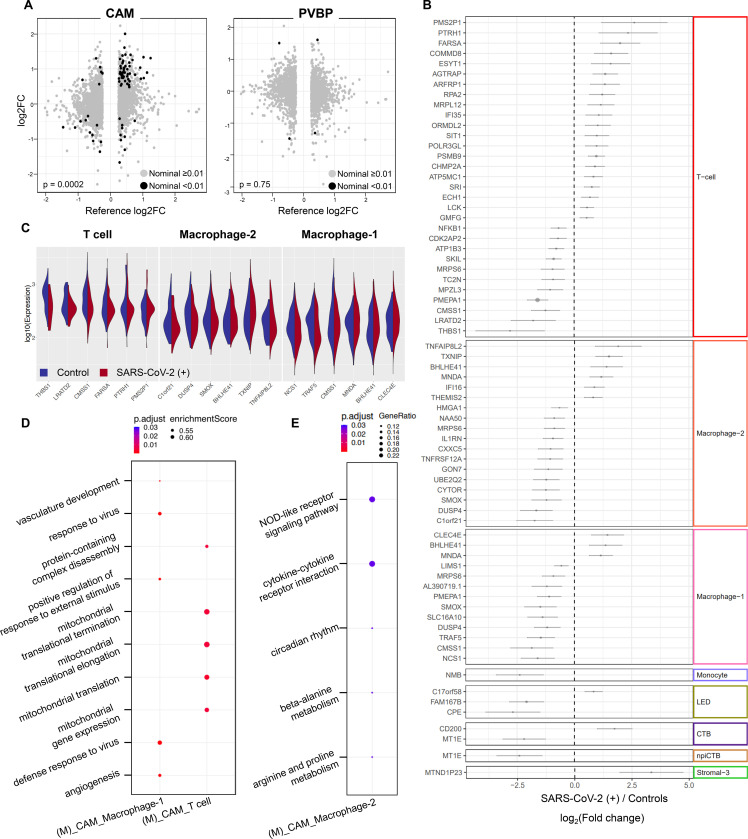
Single-cell characterization of T cells and macrophages from the chorioamniotic membranes (CAM) and placental villi and basal plate (PVBP). **(A)** Scatter plots showing the effects of SARS-CoV-2 on gene expression [log2 Fold Change (FC)] in T cells from the CAM and PVBP compared to a previously reported dataset ^40^. Black dots represent genes with nominal p < 0.01 in this study, which are used to calculate Spearman’s correlation. **(B)** Forest plot showing differentially expressed genes in T cell, macrophage-2, macrophage-1, monocyte, lymphoid endothelial decidual cell (LED), cytotrophoblast (CTB), non-proliferative interstitial cytotrophoblast (npiCTB), and stromal-3 cell populations in the CAM and PVBP of SARS-CoV-2 (+) or healthy pregnant women (n = 7–8 per group). Differentially expressed genes shown are significant after false discovery rate (FDR) adjustment (q < 0.1). **(C)** Violin plot showing the distribution of single-cell gene expression levels for the top three upregulated and downregulated genes in the maternal T cell, macrophage-1, and macrophage-2 populations in the CAM comparing between SARS-CoV-2 (+) and healthy pregnant women (n = 7–8 per group). **(D)** Gene ontology (GO) terms enriched in differentially expressed genes in the macrophage-1 and T cell populations of maternal (M) origin from CAM samples. GO terms with q < 0.05 are shown. **(E)** Kyoto Encyclopedia of Gene and Genomes (KEGG) pathways enriched for differentially expressed genes in macrophage-2 of maternal (M) origin from the CAM based on the over-representation analysis. KEGG pathways with q < 0.05 were selected.

The effects of SARS-CoV-2 on gene expression in maternal T cells from the CAM and PVBP were compared to those from peripheral T cells from hospitalized COVID-19 patients^40^, which we will refer to as the reference database hereafter. Maternal T-cell gene expression changes resulting from SARS-CoV-2 infection in the CAMs were positively correlated with those in the reference database (T cells from patients with COVID-19) (Spearman’s ρ = 0.40, p = 0.0002; Fig. 5A), suggesting a significant degree of shared DEGs. Yet, maternal T-cell gene expression induced by SARS-CoV-2 in the CAM was also distinct, since 21 out of the 31 identified DEGs were not found in the reference database. In contrast, maternal T-cell gene expression dysregulation in the PVBP was not correlated with that from the reference database (Spearman’s ρ not significantly different from 0, p = 0.75; Fig. 5A). Enrichment analysis revealed that the shared DEGs between maternal T cells in the CAMs and the reference T-cell data included translational termination and elongation, mitochondrial translational termination and elongation, and regulation of TGFβ receptor signaling (Supplementary Fig. 5A&B).

Although most of the DEGs were detected in the maternal T cells in the CAM, maternal macrophages and other cell types such as maternal monocytes, maternal LED, fetal trophoblast cell types, and fetal stromal cells also contributed to the differential gene expression observed between SARS-CoV-2 cases and controls (Fig. 5B). The top upregulated and downregulated genes in maternal T cells and macrophages are also displayed in Fig. 5C showing that changes in gene expression were not always homogeneous across all the cells (e.g., *FARSA* in T cells, and *TRAF5* in macrophages). Gene set enrichment analysis of the DEGs in maternal T cells and macrophages type 1 using Gene Ontology (GO) terms revealed that mitochondrial translational processes as well as defense response to virus and angiogenesis are processes enriched in the placental tissues from mothers infected with SARS-CoV-2 (Fig. 5D). Over-representation analysis using the DEGs in maternal macrophage type 2 revealed significant KEGG pathways including the NOD-like receptor signaling pathway and cytokine-cytokine receptor interactions (Fig. 5E). Lastly, STRING enrichment analysis of all DEGs in the CAM and PVBP showed that the interactions between GO terms including cytosol, DNA replication factor A complex, ESCRT III complex, I-kappa B/NF-kappaB complex, proteasome core complex, and alpha-subunit complex are enriched in the placental tissues of women with SARS-CoV-2 infection (Supplementary Fig. 6A).

Taken together, these data show that placentas from women with SARS-CoV-2 display alterations in their immune repertoire, mainly in maternal T cells and macrophages infiltrating the gestational tissues surrounding the fetus during gestation. Yet, the effect of SARS-CoV-2 in the fetal immune cell types is minimal in our largely asymptomatic population.

### SARS-CoV-2 RNA and proteins are not detected in the placentas of infected women

SARS-CoV-2 induced altered maternal T cell and macrophage responses in the CAM; therefore, we explored whether this virus was present in the placental tissues. First, using a scRNAseq approach, Viral-Track^41^, we explored whether viral sequences were detected in the scRNAseq data of CAMs and PVBP from women with SARS-CoV-2 infection. SARS-CoV-2 viral sequences were detected in positive controls (bronchoalveolar lavage of patients infected with SARS-CoV-2^41^) but not in the placental tissues from women with SARS-CoV-2 infection (Supplementary Fig. 6B&C).

Subsequently, we investigated the presence of viral RNA in the CAM, basal plate (BP), and placental villi (PV) using RT-qPCR for the N1 and N2 viral genes (Supplementary Fig. 7A). SARS-CoV-2 N1 and N2 proteins were not detected in any of the placental samples from women with SARS-CoV-2 infection or healthy controls (Supplementary Fig. 7B). Yet, in the spike-in positive control, N1 and N2 RNA was detected in the CAM, BP, and PV. A sensitivity assay revealed that 10 is the minimum confident copy number of viral particles detectable in the placental villi using RT-qPCR (Supplementary Fig. 7C).

**Figure 6.**
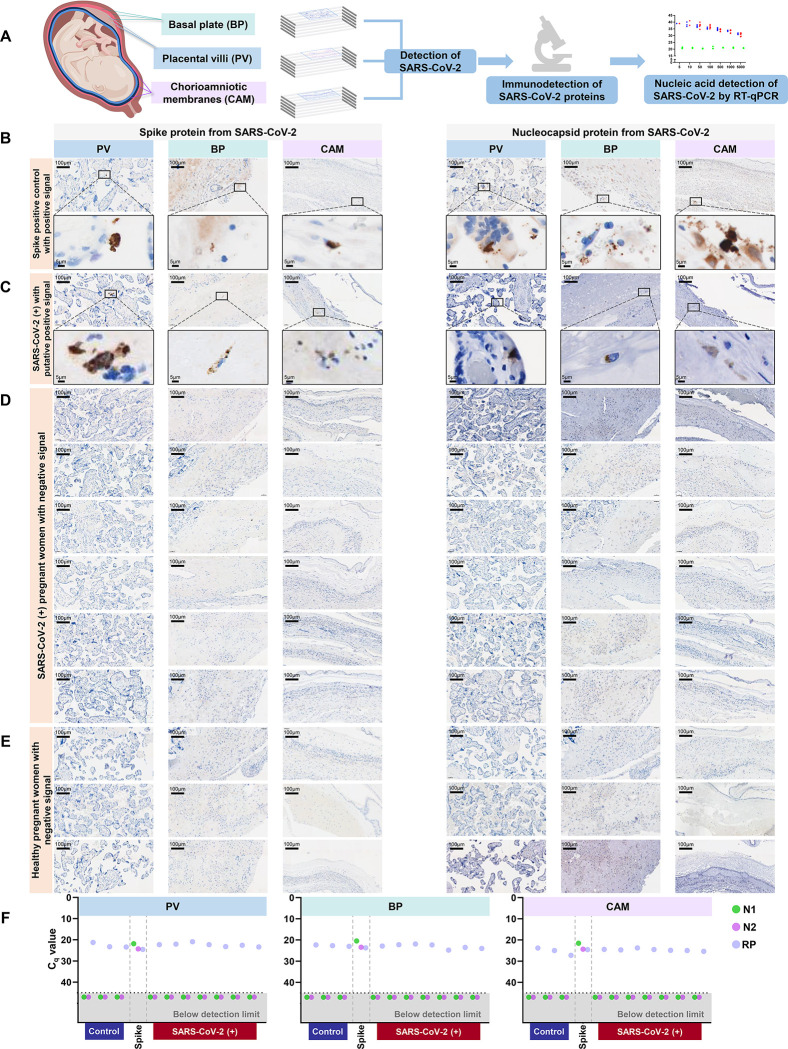
Immunohistological and molecular detection of SARS-CoV-2 proteins/RNA in the placenta of women with SARS-CoV-2 infection. **(A)** Schematic representation showing various sampling locations in the placental villi (PV), basal plate (BP), and chorioamniotic membranes (CAM) that were tested for SARS-CoV-2 proteins/RNA by immunohistochemistry and RT-qPCR, respectively. **(B)** Brightfield microscopy images showing positive signal for SARS-CoV-2 spike (left panel) and nucleocapsid (right panel) proteins in the PV, BP, and CAM of spike-in positive control. Brown color indicates putative positive staining. **(C)** Brightfield microscopy images showing putative positive signal for SARS-CoV-2 spike (left panel) and nucleocapsid (right panel) proteins in the PV, BP, and CAM of a SARS-CoV-2 (+) pregnant woman. **(D)** Brightfield microscopy images showing negative signal for SARS-CoV-2 spike (left panel) and nucleocapsid (right panel) proteins in the PV, BP, and CAM of SARS-CoV-2 (+) pregnant women. **(E)** Brightfield microscopy images showing negative signal for SARS-CoV-2 spike (left panel) and nucleocapsid (right panel) proteins in the PV, BP, and CAM of healthy pregnant women. **(F)** RT-qPCR results of SARS-CoV-2 viral RNA detection in the PV, BP, and CAM from formalin-fixed paraffin-embedded tissues from SARS-CoV-2 (+) and healthy pregnant women. N1 and N2 denote two SARS-CoV-2 nucleocapsid (N) genes, and RP denotes RNase P gene, which serves as a positive internal PCR control. Spike-in positive controls are also included. Undetermined quantification cycle (C_q_) values are represented below the detection limit (gray area).

Next, we determined whether the spike and nucleocapsid proteins were detected in the placental tissues of women with SARS-CoV-2 infection using immunohistochemistry (Fig. 6A). Several histological slides from the CAM, BP, and PV were included in our evaluation, including negative and spike-in positive controls (Supplementary Table 4). Both SARS-CoV-2 spike and nucleocapsid proteins were identified in the spike-in positive controls in the CAM, PB, and BP (Fig. 6B). A few of the placentas from asymptomatic women with SARS-CoV-2 infection displayed a putative positive signal for the spike and nucleocapsid proteins (Fig. 6C); yet, in all other cases, the placental tissues were negative for the SARS-CoV-2 proteins (Fig. 6D). As expected, spike and nucleocapsid SARS-CoV-2 proteins were not detected in the placental tissues of control women (Fig. 6E). To verify the detection of SARS-CoV-2 in the placental tissues, RNA was isolated from the same FFPE tissue sections where the putative positive signals were observed and RT-qPCR for the N1 and N2 viral genes was performed. FFPE tissue sections from the placental tissues of control women and spike-in positive controls were also included. None of the placentas from women with SARS-CoV-2 infection or controls had detectable levels of N1 and N2 RNA viral genes; yet, the spike-in positive controls were detected (Fig. 6F).

Collectively, these data show that SARS-CoV-2 is not detected in the placental tissues, including the chorioamniotic membranes, of women infected with SARS-CoV-2.

### SARS-CoV-2 infection during pregnancy does not compromise the sterility of the placenta

**Figure 7.**
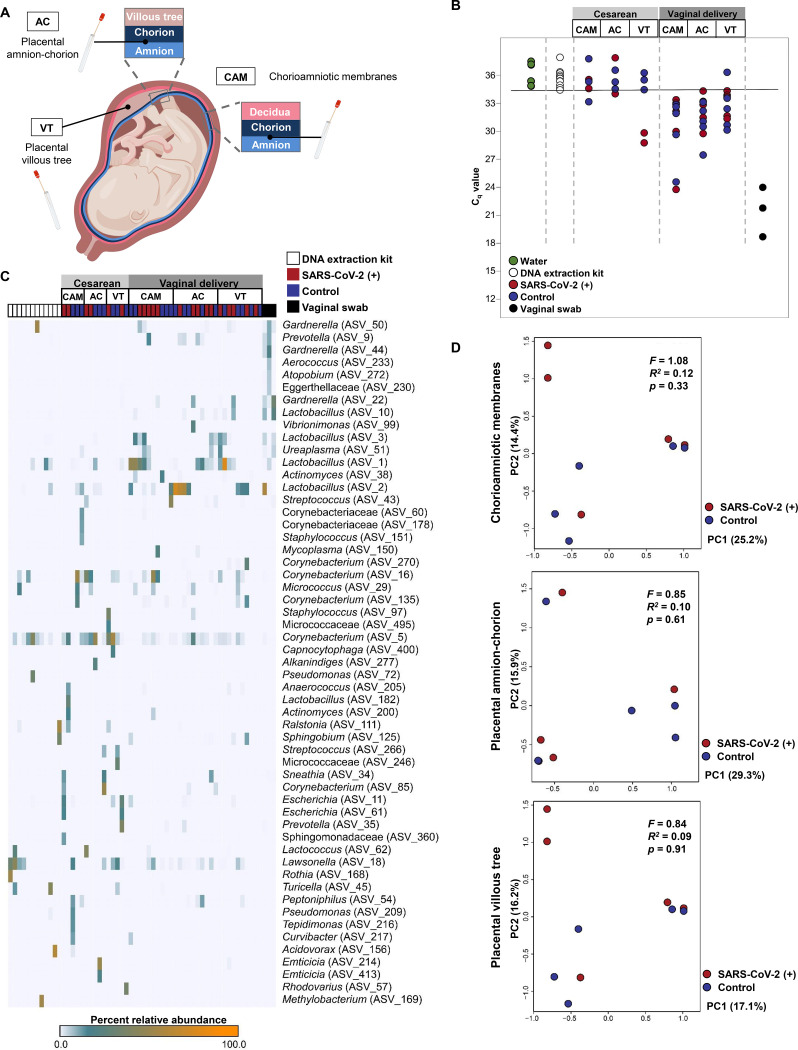
Bacterial DNA profiles of the placental tissues from pregnant women with SARS-CoV-2 infection. **(A)** Schematic representation of sampling locations from the chorioamniotic membranes (CAM), amnion-chorion interface of the placenta (AC), and within the placental villous tree (VT) from SARS-CoV-2 (+) women who delivered by cesarean section (n = 2) or vaginally (n = 5) and from healthy pregnant women who delivered by cesarean section (n = 3) or vaginally (n = 5). **(B)** Quantitative real-time PCR analyses illustrating the bacterial loads (i.e. 16S rDNA abundance) of the CAM, AC, and VT from SARS-CoV-2 (+) or healthy pregnant women (cesarean section or vaginal delivery). The solid black line denotes the lowest cycle of quantification (i.e. highest bacterial load) for any blank DNA extraction kit negative control. Data from three human vaginal swabs are included for perspective. **(C)** Heatmap illustrating the relative abundances of prominent (>2% average relative abundance) amplicon sequence variants (ASVs) among the 16S rRNA gene profiles of the CAM, AC, and VT from SARS-CoV-2 (+) or healthy pregnant women (cesarean section or vaginal delivery). Data from blank DNA extraction kit negative controls and human vaginal swabs are included for perspective. **(D)** Principal coordinates analyses (PCoA) illustrating similarity in the 16S rRNA gene profiles of the CAM, AC, and VT obtained through vaginal delivery from SARS-CoV-2 (+) or healthy pregnant women.

Lastly, we investigated whether SARS-CoV-2 infection during pregnancy affected the molecular microbial profiles of the placental tissues, including the chorioamniotic membranes. Specifically, we used 16S rRNA gene qPCR and sequencing to characterize the bacterial DNA load and profiles of the amnion-chorion interface of the extraplacental chorioamniotic membranes, the amnion-chorion interface of the placental disc, and the placental villous tree (Fig. 7A). Mode of delivery was the principal factor affecting bacterial DNA load (Supplementary Table 5) and profile. Very few samples (4/15) from cesarean deliveries had a bacterial DNA load exceeding that of technical controls for background DNA contamination (i.e. blank DNA extraction kits), yet almost all of the samples (29/30) from vaginal deliveries did (Fig. 7B). Furthermore, whereas the bacterial DNA profiles of samples from cesarean deliveries were similar to those of technical controls, those from vaginal deliveries were distinct, being dominated by DNA signals from *Lactobacillus* and *Ureaplasma,* similar to the vaginal swab positive controls (Fig. 7C). Among the samples obtained from vaginal deliveries, there was no difference in the bacterial DNA profiles based on maternal SARS-CoV-2 infection status (Fig. 7D). These findings show that, although mode of delivery alters the bacterial DNA loads and profiles of the placental tissues, we did not find evidence that the same is true for maternal SARS-CoV-2 infection.

## DISCUSSION

This study provides evidence that, in a largely asymptomatic population, SARS-CoV-2 infection in pregnancy is primarily associated with maternal inflammatory responses in the circulation and at the maternal-fetal interface. First, we showed that pregnant women with SARS-CoV-2 infection had elevated levels of IgM and IgG in the peripheral circulation, whereas only IgG was detectable in the cord blood of their neonates, suggesting that acute fetal infection did not occur. This finding is consistent with several reports showing that IgM is undetected in the cord blood of neonates born to women with SARS-CoV-2 infection^29,42,43^. However, few studies have demonstrated that both IgM and IgG are detectable in a small fraction of neonates born to women diagnosed with COVID-19^16,24,30^. The increased levels of IgG in the cord blood are explained by the fact that this immunoglobulin crosses the placenta via the neonatal Fc receptor (nFcR), which is highly expressed in the syncytiotrophoblast layer^44,45^. Yet, it has been recently reported that, in the third trimester, the mechanisms whereby SARS-CoV-2-specific IgG1 crosses the placenta are compromised due to altered glycosylation profiles^46^. In contrast, IgM cannot cross the placenta due to its large molecular weight, and thus the detection of this immunoglobulin in the cord blood represents an acute fetal response in the clinical setting^47,48^. Therefore, the absence of detectable IgM in the cord blood suggests that vertical transmission *in utero* of SARS-CoV-2 was unlikely to occur in our study population.

In the current study, we report that pregnant women mount a mild systemic inflammatory response to SARS-CoV-2, which is consistent with observations in asymptomatic non-pregnant individuals with SARS-CoV-2 infection^49^. Interestingly, we found that neonates born to SARS-CoV-2-infected mothers also demonstrated increased levels of cytokines such as IL-17A and TNF in the cord blood. IL-17A is a pro-inflammatory cytokine associated with a hyper-inflammatory state and severe immunopathologies^50^, including COVID-19^51^. Indeed, the severity of COVID-19 was associated with increasing systemic levels of IL-17A or Th17-like cells^32,52,53^, and its inhibition has been proposed as a potential treatment for this disease^54^. TNF is a stereotypical pro-inflammatory cytokine implicated in a plethora of physiological and pathological processes^55^. This cytokine is positively correlated with SARS-CoV-2 viral load^32^ and the severity of COVID-19 disease^53^. Moreover, an inverse relationship exists between TNF levels and total T-cell counts in COVID-19 patients^56^. Taken together, these data indicate that SARS-CoV-2 infection not only causes a maternal cytokine response but may also induce fetal inflammation, despite the absence of detectable IgM in the cord blood. Alternatively, the increased concentrations of some cytokines (e.g. IL-8) in the cord blood could be explained by transfer of maternal cytokines through the placental tissues^57,58^. However, the mechanisms whereby maternal SARS-CoV-2 infection may elicit fetal cytokine responses require further investigation.

Importantly, we also report that neonates born to women with SARS-CoV-2 infection had low concentrations of IL-6 in the cord blood. Interleukin-6 is a pleiotropic cytokine, which functions range from hematopoiesis to metabolic regulation of inflammation, autoimmunity, and acute phase response^59^. In viral infections, IL-6 can display pathogenic or protective effects *in vivo*^60^, which resembles the functions of this cytokine in pregnancy^61^. Consistently, elevated systemic IL-6 levels in patients with SARS-CoV-2 infection are considered to have predictive value for disease severity^62^. In contrast, low levels of this cytokine are associated with good prognosis^63^. Thus, we suggest that neonates born to asymptomatic pregnant women with SARS-CoV-2 infection display reduced concentrations of IL-6 as a compensatory mechanism to prevent further acute inflammation.

A hallmark of SARS-CoV-2 infection is lymphopenia, which is primarily reflected in the T-cell compartment^53,64–68^, but not consistently observed for B cells^69^. Specifically, patients with symptomatic COVID-19 displayed reduced numbers of CD4+ and CD8+ T-cell subsets including naïve, central memory, and effector memory cells^34,66,68,70,71^. Lymphopenia is also correlated with COVID-19 disease severity, as critically ill patients showed the lowest numbers of total lymphocytes, including T-cells, compared to asymptomatic individuals^72^. Yet, asymptomatic or mildly ill pregnant women seem to have slightly reduced lymphocyte numbers when compared to healthy controls^73^. Indeed, a recent single-center study showed that 80% of pregnant women with mild or asymptomatic SARS-CoV-2 infection displayed lymphopenia^74^. Consistently, we found that pregnant women with SARS-CoV-2 infection had reduced T-cell numbers compared to healthy controls, which included specific subsets such as CD4^+^ T_CM_, Th1-like, CD8^+^ T_EM_, and Tc17-like cells. Both Th1 and Tc17 cells participate in orchestrating pro-inflammatory responses in health and disease^75,76^. During pregnancy, these T-cell subsets are implicated in the establishment and maintenance of maternal-fetal tolerance^77–79^, which play a central role in pregnancy success^80–90^. Hence, these results indicate that SARS-CoV-2 infection alters specific pro-inflammatory T-cell subsets in the maternal circulation, which may compromise the mechanisms of maternal-fetal tolerance.

Concurrent with the cellular immune changes occurring in the periphery of pregnant women with SARS-CoV-2 infection, maternal T-cell responses in the chorioamniotic membranes were also altered, as revealed by our scRNAseq data. Maternal T cells reside at the maternal-fetal interface and their abundance changes as gestation progresses^79,91^. This T-cell compartment comprises multiple subsets, including effector/activated T cells, regulatory T cells, and exhausted T cells^78,92,93^. In addition, these adaptive immune cells can participate in the processes of labor by releasing inflammatory mediators such as TNF, IL-1β, and MMP-9^94^. The importance of T cells in the process of labor is underscored by observations showing that their single-cell signatures can be detected in the maternal circulation, providing a non-invasive approach to monitor pregnancy and its complications^39,95^. Consistent with these findings, herein we demonstrated that the single-cell signature of maternal T cells in the chorioamniotic membranes from SARS-CoV-2-infected pregnant women resembled that of peripheral T cells from non-pregnant infected patients (obtained from a previously reported dataset^40^). These results suggest that both systemic and local T-cell responses are altered by SARS-CoV-2; yet, pregnancy also promotes stereotypical cellular responses. Interestingly, maternal T cells from the chorioamniotic membranes displayed enrichment of gene ontology terms related to mitochondrial gene expression and translation, a process that has been implicated in T-cell functions including cytokine production^96^. Therefore, SARS-CoV-2 may enhance maternal T-cell function at the maternal-fetal interface.

In the current study, SARS-CoV-2 infection also had effects on maternal macrophages in the chorioamniotic membranes. The processes and pathways enriched in these tissue-resident innate immune cells included response to virus, NOD-like receptor signaling pathway, and cytokine-cytokine receptor interaction, highlighting the role of macrophages in the host response against SARS-CoV-2 infection^97–99^. Other processes enriched in maternal macrophages included vasculature development and angiogenesis, supporting a role for these cells in the vascular damage to the placentas of women with COVID-19^25^. Thus, maternal macrophage responses may act as a double-edged sword in the chorioamniotic membranes of women with SARS-CoV-2 infection by modulating host immune responses while simultaneously contributing to placental vasculopathy.

Importantly, we report that SARS-CoV-2 infection during pregnancy was neither associated with alterations in the neonatal T-cell repertoire nor with fetal immune responses in the placenta. These observations are in tandem with the absence of SARS-CoV-2 transcripts/proteins in the placenta and chorioamniotic membranes as well as undetectable IgM in the cord blood. Our results are in agreement with numerous reports showing that SARS-CoV-2 is undetected in the placenta^24,100,101^, amniotic fluid^102–104^, and neonates^5,24,29,102,103^. Yet, SARS-CoV-2 has been reported in the placentas of severe COVID-19 patients^15,16,23,27,105^, indicating that this virus can on rare occasions reach and infect this organ. Therefore, the absence of SARS-CoV-2 in the chorioamniotic membranes, placental villi, and basal plate of our mostly asymptomatic study population is in accordance with the known scarcity of placental infection ^106^.

Traditionally, the placenta is considered a sterile organ^107,108^. Indeed, recent research has reiterated the sterile womb hypothesis using placentas from women who delivered via cesarean section at term without labor^109–111^ as well as studies in mice^112,113^ and non-human primates^114^. Here, we evaluated the possibility that maternal SARS-CoV-2 infection compromises the sterility of the placenta by facilitating the invasion of bacteria or the transfer of bacterial DNA from maternal compartments. Consistent with our previous studies^109^, the placentas of women who delivered via cesarean section did not consistently harbor a microbiome. Women who delivered vaginally displayed placental bacterial signatures similar to those from the lower genital tract; yet, maternal SARS-CoV-2 infection did not modify such signatures. Hence, SARS-CoV-2 infection does not affect placental sterility in mostly asymptomatic women who delivered a term neonate.

In summary, we have shown that SARS-CoV-2 infection during pregnancy primarily induces specific maternal inflammatory responses in the periphery and at the maternal-fetal interface, the latter being governed by T cells and macrophages. Maternal SARS-CoV-2 infection was also associated with a cytokine response in the neonatal circulation without compromising the cellular immune repertoire. Moreover, SARS-CoV-2 infection during pregnancy neither altered fetal inflammatory responses in the placenta nor induced elevated levels of IgM in the cord blood. Importantly, SARS-CoV-2 was not detected in the placentas of infected women, nor was the sterility of the placenta compromised by this virus. This study provides insight into the maternal-fetal immune responses triggered by SARS-CoV-2 and further emphasizes the rarity of placental infection.

## METHODS

### Human subjects, clinical specimens, and definitions

Human maternal peripheral blood, umbilical cord blood, and placental tissues, including chorioamniotic membrane samples, were obtained at the Perinatology Research Branch, an intramural program of the *Eunice Kennedy Shriver* National Institute of Child Health and Human Development (NICHD), National Institutes of Health, U.S. Department of Health and Human Services, Wayne State University (Detroit, MI, USA), and the Detroit Medical Center (DMC) (Detroit, MI, USA). The collection and use of human materials for research purposes were approved by the Institutional Review Boards of Wayne State University School of Medicine, Detroit Medical Center, and NICHD. All participating women provided written informed consent prior to sample collection. The study groups were divided into pregnant women who had a positive RT-PCR test for SARS-CoV-2 (nasopharyngeal test provided by the Detroit Medical Center) and healthy gestational age-matched controls. The demographic and clinical characteristics of the study groups are shown in Supplementary Table 1. The maternal peripheral blood was collected at admission, prior to the administration of any medication, and the umbilical cord blood and placental tissues were collected immediately after delivery.

Gestational age was established based on the last menstrual period and confirmed by ultrasound examination. Labor was defined as the presence of regular uterine contractions with a frequency of ≥2 times every 10 minutes and cervical ripening. Term delivery was defined as birth ≥37 weeks of gestation. Preeclampsia was defined as new-onset hypertension that developed ≥20 weeks of gestation and proteinuria^115^. Other clinical and demographic characteristics were obtained by review of medical records.

### Placental histopathological examination

Placentas were examined histologically by perinatal pathologists according to standardized DMC protocols^116^. Briefly, three to nine sections of the placenta were examined, and at least one full-thickness section was taken from the center of the placenta; others were taken randomly from the placental disc. Acute and chronic inflammatory lesions of the placenta (maternal inflammatory response and fetal inflammatory response), as well as other placental lesions were diagnosed according to established criteria^116–120^, as shown in Supplementary Table 1.

### Immunoassays

#### Immunoglobulin (Ig) M and G determination in the maternal blood and umbilical cord blood

Maternal peripheral blood and umbilical cord blood was collected into tubes without an anticoagulant, and the tubes were stored at room temperature for 30–60 minutes prior to centrifugation for 10 min at 1,600 x g and 4°C. After centrifugation, the serum was collected and stored at −80°C. The serum concentrations of SARS-CoV-2 IgM and IgG were determined using the human anti-SARS-CoV-2 IgM and human anti-SARS-CoV-2 IgG ELISA kits (LifeSpan BioSciences, Inc., Seattle, WA, USA), according to the manufacturer’s instructions. Plates were read using the SpectraMax iD5 (Molecular Devices, San Jose, CA, USA) and analyte concentrations were calculated with the SoftMax Pro 7 (Molecular Devices). The sensitivities of the assays were 0.469 ng/mL (human anti-SARS-CoV-2 IgM) and 2.344 ng/mL (human anti-SARS-CoV-2 IgG).

#### Determination of cytokine and chemokine concentrations in the maternal blood and umbilical cord blood

Maternal peripheral blood and umbilical cord blood was collected into tubes with an anticoagulant (EDTA or citrate), which were centrifuged for 10 min at 1,600 x g and 4°C. Upon centrifugation, the plasma was collected and stored at −80°C prior to cytokine/chemokine determination. The V-PLEX Pro-Inflammatory Panel 1 (human) and Cytokine Panel 1 (human) immunoassays (Meso Scale Discovery, Rockville, MD, USA) were used to measure the concentrations of IFN-γ, IL-1β, IL-2, IL-4, IL-6, IL-8, IL-10, IL-12p70, IL-13, and TNF (Pro-inflammatory Panel 1) or GM-CSF, IL-1α, IL-5, IL-7, IL-12/IL-23p40, IL-15, IL-16, IL-17A, TNF-β, and VEGF-A (Cytokine Panel 1) in the maternal and cord blood plasma, according to the manufacturer’s instructions. Plates were read using the MESO QuickPlex SQ 120 (Meso Scale Discovery) and analyte concentrations were calculated with the Discovery Workbench 4.0 (Meso Scale Discovery). The sensitivities of the assays were: 0.21–0.62 pg/mL (IFN-γ), 0.01–0.17 pg/mL (IL-1β), 0.01–0.29 pg/mL (IL-2), 0.01–0.03 pg/mL (IL-4), 0.05–0.09 pg/mL (IL-6), 0.03–0.14 pg/mL (IL-8), 0.02–0.08 pg/mL (IL-10), 0.02–0.89 pg/mL (IL-12p70), 0.03–0.73 pg/mL (IL-13), 0.01–0.13 pg/mL (TNF), 0.08–0.19 pg/mL (GM-CSF), 0.05–2.40 pg/mL (IL-1α), 0.04–0.46 pg/mL (IL-5), 0.08–0.17 pg/mL (IL-7), 0.25–0.42 pg/mL (IL-12/IL-23p40), 0.09–0.25 pg/mL (IL-15), 0.88–9.33 pg/mL (IL-16), 0.19–0.55 pg/mL (IL-17A), 0.04–0.17 pg/mL (TNF-β), 0.55–6.06 pg/mL (VEGF-A).

#### Immunophenotyping of maternal and cord blood leukocytes

Maternal peripheral blood and umbilical cord blood was collected into tubes containing EDTA. Fifty μL of whole blood were incubated with fluorochrome-conjugated anti-human mAbs (Supplementary Table 6) for 30 min at 4°C in the dark. After incubation, erythrocytes were lysed using BD FACS lysing solution (BD Biosciences, San Jose, CA, USA). The resulting leukocytes were washed and resuspended in 0.5 mL of FACS staining buffer (BD Biosciences) and acquired using the BD LSRFortessa flow cytometer and FACSDiva 6.0 software. The absolute number of cells was determined using CountBright absolute counting beads (Thermo Fisher Scientific/Molecular Probes, Eugene, OR, USA). The analysis and figures were performed using the FlowJo software version 10 (FlowJo, Ashland, OR, USA). Immunophenotyping included the identification of: general leukocyte populations (neutrophils, monocytes, T cells, B cells, and NK cells), monocyte subsets, neutrophil subsets, T-cell subsets, and B-cell subsets. Specifically, the numbers of effector memory T cells (T_EM_; CD3^+^CD4^+^/CD8^+^CD45RA^−^CCR7^−^), naïve T cells (T_N_; CD3^+^CD4^+^/CD8^+^CD45RA^+^CCR7^+^), central memory T cells (T_CM_; CD3^+^CD4^+^/CD8^+^CD45RA^−^CCR7^+^), terminally-differentiated effector memory T cells (T_EMRA_; CD3^+^CD4^+^/CD8^+^CD45RA^+^CCR7^−^), Th1/Tc1-like T cells (CD3^+^CD4^+^/CD8^+^CXCR3^+^CCR6^+^/CCR6^−^), Th2/Tc2-like T cells (CD3^+^CD4^+^/CD8^+^CXCR3^−^CCR6^−^), and Th17/Tc17-like T cells (CD3^+^CD4^+^/CD8^+^CXCR3CCR6^+^) in maternal and cord blood are presented in Fig. 3.

#### Reactive oxygen species (ROS) production by neutrophils and monocytes

Fifty μL of maternal peripheral blood and cord blood were stimulated with 50 μL of ROS assay mix containing 1:250 of ROS assay stain and ROS assay buffer [both from the ROS assay Kit (eBioscience, San Diego, CA, USA)], and 1 μL of phorbol myristate acetate (PMA; 3 μg/mL) (Millipore Sigma, Burlington, MA, USA). The unstimulated group received 1:250 ROS assay mix and 1X phosphate buffered saline (PBS) (Thermo Fisher Scientific/Gibco, Grand Island, NY, USA). The cells were incubated at 37°C with 5% CO_2_ for 60 min. Following incubation, erythrocytes were lysed using Ammonium-Chloride-Potassium (ACK) lysing buffer (Lonza, Walkersville, MD, USA), and the resulting leukocytes were collected after centrifugation at 300 x g for 5 min. Next, leukocytes were resuspended in 0.5 mL of 1X PBS and acquired using the BD LSRFortessa flow cytometer and FACSDiva 6.0 software to measure ROS production by neutrophils and monocytes. The analysis and figures were performed using the FlowJo software version 10.

### Single-cell RNA sequencing

#### Preparation of single-cell suspensions

Single-cell suspensions were prepared from the basal plate, placental villi, and chorioamniotic membranes, as previously described with modifications^39^. Digestion of placental tissues was performed using collagenase A (Sigma Aldrich, St. Louis, MO, USA) or the enzyme cocktail from the Umbilical Cord Dissociation Kit (Miltenyi Biotec, San Diego, CA, USA). Next, tissue suspensions were washed with 1X PBS and filtered through a cell strainer (Miltenyi Biotec). Cell pellets were collected after centrifugation at 300 x g for 10 min at 20°C. Erythrocytes were lysed using ACK lysing buffer and the reaction was stopped by washing with 0.04% Bovine Serum Albumin (BSA) (Sigma Aldrich) in 1X PBS. Then, the cell pellets were collected after centrifugation at 300 x g for 10 min at 20°C and resuspended in 1X PBS for cell counting using an automatic cell counter (Cellometer Auto 2000; Nexcelom Bioscience, Lawrence, MA). Dead cells were removed from the cell suspensions using the Dead Cell Removal Kit (Miltenyi Biotec) to obtain a final cell viability of ≥80%.

#### Single-cell library preparation using the 10x Genomics platform

Viable cells were used for single-cell RNAseq library preparation following the protocol for the 10x Genomics Chromium Single Cell 3’ Gene Expression Version 3 Kit (10x Genomics, Pleasanton, CA, USA). Briefly, cell suspensions were loaded into the Chromium Controller to generate gel beads in emulsion (GEM), each containing a single cell and a single Gel Bead with barcoded oligonucleotides. Reverse transcription of mRNA into complementary (c)DNA was performed using the Veriti 96-well Thermal Cycler (Thermo Fisher Scientific, Wilmington, DE, USA). The resulting cDNA was purified using Dynabeads MyOne SILANE (10x Genomics) and the SPRIselect Reagent (Beckman Coulter, Indianapolis, IN, USA). cDNA amplicons were optimized via enzymatic fragmentation, end-repair, and A-tailing followed by the incorporation of adaptors and sample index by ligation. The sample index PCR product was amplified using the Veriti 96-well Thermal Cycler. The Agilent Bioanalyzer High Sensitivity Chip (Agilent Santa Clara, CA, USA) was used to analyze and quantify the final library construct. The Kapa DNA Quantification Kit for Illumina platforms (Kapa Biosystems, Wilmington, MA, USA) was used to quantify the DNA libraries, following the manufacturer’s instructions.

#### Sequencing

10x scRNAseq libraries were sequenced on the Illumina NextSeq 500 in the Genomics Services Center (GSC) of the Center for Molecular Medicine and Genetics (Wayne State University School of Medicine, Detroit, MI, USA). The Illumina 75 Cycle Sequencing Kit (Illumina, San Diego, CA, USA) was used with 58 cycles for R2, 26 for R1, and 8 for I1.

#### Genotyping

DNA was extracted from maternal peripheral blood and umbilical cord blood/tissue using DNeasy Blood and Tissue Kit (Qiagen, Hilden, Germany), following manufacturer’s instructions modified with the addition of 4 μl RNase A (100 mg/mL) (Qiagen) and incubation in 56°C. Purified DNA samples were quantified using Qubit™ dsDNA HS Assay Kit (Invitrogen, Carlsbad, CA, USA). Two platforms were used for genotyping: i) low-coverage (~0.4X) whole-genome sequencing imputed to 37.5 M variants using the 1000 Genomes database (Gencove, New York, NY, USA); and ii) Infinium Global Diversity Array-8 v1.0 Kit microarrays processed by the Advanced Genomics Core of University of Michigan (Ann Arbor, MI, USA). For the array platform, genotype information was converted to vcf format using “iaap-cli gencall” and “gtc_to_vcf.py” from Illumina, and imputation to 37.5 M variants using the 1000 Genomes haplotype references was done using the University of Michigan Imputation Server (https://imputationserver.sph.umich.edu/). The maternal/fetal relationship of the genotyped samples was ascertained using plink2 KING-robust kinship analysis^121^. The vcf files from the two platforms were then merged together and filtered for high quality imputation and coverage for at least ten scRNAseq transcripts using bcftools.

#### scRNAseq data analysis

Sequencing data were processed using Cell Ranger version 4.0.0 from 10x Genomics for de-multiplexing. The fastq files were then aligned using kallisto^122^, and bustools^123^ summarized the cell/gene transcript counts in a matrix for each sample using the “lamanno” workflow for scRNAseq. Each library was then processed using DIEM^124^ to eliminate debris and empty droplets. In parallel, “cellranger counts” was also used to align the scRNAseq reads using the STAR^125^ aligner to produce the bam files necessary for demultiplexing the individual of origin, based on the genotype information using souporcell^126^ and demuxlet^127^. We removed any droplet/GEM barcode that was assigned to doublet or ambiguous cells in demuxlet or souporcell, and only those cells that could be assigned a pregnancy case and maternal/fetal origin were kept. All count data matrices were then normalized and combined using the “NormalizeData,” “FindVariableFeatures,” and “ScaleData” methods implemented in the Seurat package in R (Seurat version 3.1, R version 4.0.0)^128,129^. Next, the Seurat “RunPCA” function was applied to obtain the first 100 principal components, and the different libraries were integrated and harmonized using the Harmony package in R version 1.0^130^. The top 30 harmony components were then processed using the Seurat “runUMAP” function to embed and visualize the cells in a two-dimensional map via the Uniform Manifold Approximation and Projection for Dimension Reduction (UMAP) algorithm^131,132^. To label the cells, the SingleR^133^ package in R version 1.3.8 was used to assign a cell-type identity based on our previously labeled data as reference panel (as performed in^39^). Cell type abbreviations used are: STB, syncytiotrophoblast; EVT, extravillous trophoblast; CTB, cytotrophoblast; npiCTB, non-proliferative interstitial cytotrophoblast; LED, lymphoid endothelial decidual cell; and NK, natural killer cell.

#### Differential gene expression

To identify differentially expressed genes, we created a pseudo-bulk aggregate of all the cells of the same cell-type/location/origin. For each combination, we only used samples with more than 20 cells. The negative binomial model implemented in the DESeq2 R package version 1.28.1^134^ was used to calculate the log_2_ fold change (FC) between SARS-CoV-2 (+) and healthy pregnant women. The p-values were adjusted using the false discovery rate method (FDR)^135^, and the DEGs were selected based on an adjusted p-value < 0.1. qq-plot was used to assess the distribution of the p-values and to identify which cell types and location combinations have higher enrichment for low p-values. Forest plots were used to visualize the DEGs, with each dot representing the log_2_FC of the SARS-CoV-2 (+) group and the bars representing the 95% confidence interval. The genes with the highest log_2_FC across T-cell, Macrophage-1, and Macrophage-2 cell types were further illustrated using violin plots representing the single-cell gene expression data in log_10_[transcripts per million (TPM)].

#### Comparison with previous scRNAseq SARS-CoV-2 studies

Single-cell RNAseq data showing the effects of SARS-CoV-2 on peripheral T cells was obtained from a previous study^40^. The log_2_FCs from this previous study were compared to those obtained here in maternal T cells from the placental villi and basal plate (PVBP) and the chorioamniotic membranes (CAM). The comparison was visualized with scatter plots using the ggplot2 R package version 3.3.2 and Spearman’s correlation analysis. Additionally, this previously generated set of SARS-CoV-2-associated genes in T cells was used to repeat the FDR p-value adjustment to reduce the burden of multiple testing in CAM-derived maternal T-cells and provide a longer list of genes. This list of genes was further analyzed with the clusterProfiler in R version 3.16.1 to perform gene set enrichment analysis (GSEA) and over-representation analysis (ORA).

#### Gene ontology and pathway enrichment analysis

The clusterProfiler in R version 3.16.1^136^ was used to perform GSEA and ORA based on the Gene Ontology (GO), Kyoto Encyclopedia of Gene and Genomes (KEGG), and Reactome databases. The ORA determines if biological pathways or processes are enriched in a list of DEGs. GSEA calculates the enrichment score (ES) for each gene set^137^ with respect to the full list of genes ranked by −log_10_(p-value). P-values were adjusted for multiple comparisons using the FDR method^135^. The functions “enrichPathway”, “enrichKEGG”, and “gseGO” from “clusterProfiler” were used to perform the ORA and GSEA analyses separately for each list of genes obtained as differentially expressed for each cell type, placental compartment, and maternal/fetal origin. Only results that were significant after correction were reported with q < 0.05 being considered statistically significant.

#### STRING Analysis

The STRING database (https://string-db.org) was utilized to identify and visualize the enrichment of GO terms among all the DEGs, regardless of cell type, compartment, and origin. The STRING database integrates the known and predicted protein-protein associations from many organisms, including both direct (physical) and indirect (functional) interactions^138^. The significant gene ontologies (cellular components) (q < 0.05) were selected and highlighted by different colors.

#### Analysis of viral reads in scRNAseq libraries

The R-based computational pipeline Viral-Track was used to study viruses in raw scRNAseq data (github.com/PierreBSC/Viral-Track)^41^. A combined index of both the host GRCH37(hg19) and viral reference genomes was constructed in Viral-Track. The viral genomes were downloaded from the Virusite database version 2020.3^139^ that includes all published viruses, viroids, and satellites (NCBI RefSeq). Afterwards, the STAR aligner was used to map reads to the indexed host and viral genome. Viral genomes were filtered based on read-map quality, nucleotide composition, sequence complexity, and genome coverage. Sequence complexity was calculated by computing the average nucleotide frequency and Shannon’s entropy. Reads with a sequence entropy above 1.2, genome coverage greater than 5%, and longest contig longer than three times the mean read length are required for a viral segment to be considered present (default thresholds empirically defined by Viral-Track). As no viral reads were detected in our PVBP and CAM libraries, the correct implementation of the Viral-Track pipeline was validated by reanalyzing the data of broncho-alveolar lavage samples of patients with severe and mild SARS-CoV-2^41^ and reproducing the detection of SARS-CoV-2 and human metapneumovirus.

### Detection of SARS-CoV-2 RNA/ proteins in the placenta

#### Detection of SARS-CoV-2 RNA in the placenta

Total RNA was isolated from the basal plate, placental villi, and chorioamniotic membranes using QIAshredders and RNeasy Mini Kit (both from Qiagen), according to the manufacturer’s instructions. Positive and negative controls were SARS-Related Coronavirus 2 (SARS-CoV-2) External Run Control and Negative Control (both from ZeptoMetrix, Buffalo, NY, USA). Following the instructions from the CDC-2019 Novel Coronavirus (2019-nCoV) Real-Time RT-PCR Diagnostic Panel, cDNA was synthesized using TaqPath™ 1-Step RT-qPCR Master Mix, CG (Thermo Fisher Scientific/Applied Biosystems, Frederick, MD, USA) and primers from the 2019-nCoV RUO Kit (Integrated DNA Technologies, Newark, NJ, USA). Reactions were incubated at 25°C for 2 min followed by 50°C for 15 mins. Initial denaturation was set for 2 min at 95°C followed by 45 amplification cycles at 95°C for 3 sec and 55°C for 30 sec. A cycle of quantification (C_q_ value) less than 45 indicates a positive result. Two positive PCR controls were used: 2019-nCoV_N (virus) and Hs_RPP30 (human) (both from Integrated DNA Technologies). Each PCR sample was run in duplicate.

RNA extractions were also performed using QIAamp Viral RNA Mini Kit (Qiagen) and results were comparable to those generated using the RNeasy Mini Kit.

#### SARS-CoV-2 Viral Particle Sensitivity Assay

For each experiment (n = 3), ten pieces of freshly collected placental villi from pregnant women were homogenized separately. Nine of the homogenates were spiked with increasing numbers of viral particles [SARS-Related Coronavirus 2 (SARS-CoV-2) External Run Control] (0 to 5,000 particles/homogenate). SARS-Related Coronavirus 2 (SARS-CoV-2) Negative Control was added to the last homogenate prior to mechanical digestion. Total RNA was isolated using the RNeasy Mini Kit, according to manufacturer’s instructions. cDNA synthesis and PCR was performed as described above.

#### Detection of SARS-CoV-2 proteins in the placenta

Five-μm-thick tissue sections of formalin-fixed, paraffin-embedded placental villi (PV), basal plate (BP), and the chorioamniotic membranes (CAM) were cut, mounted on SuperFrost™ Plus microscope slides (Erie Scientific LLC, Portsmouth, NH, USA), and subjected to immunohistochemistry using SARS-CoV/SARS-CoV-2 (COVID-19) spike antibody [1A9] (GeneTex, Irvine, CA, USA) and SARS-CoV-2 (COVID-19) nucleocapsid antibody (GeneTex). To serve as a positive control, tissues from pregnant women were spiked with SARS-CoV-2 (Isolate: USA/WA1/2020) (ZeptoMetrix) Culture Fluid (heat inactivated). Spiked tissues were subjected to immunohistochemistry using SARS-CoV/SARS-CoV-2 (COVID-19) spike antibody [1A9] and SARS-CoV-2 (COVID-19) nucleocapsid antibody. Staining was performed using the Leica Bond-Max automatic staining system (Leica Microsystems, Wetzlar, Germany) with the Bond Polymer Refine Detection Kit (Leica Microsystems). The mouse isotype (Agilent) and rabbit isotype (Agilent) were used as negative controls. Tissue slides were then scanned using the Vectra Polaris Multispectral Imaging System (Akoya Biosciences, Marlborough, MA, USA) and images were analyzed using the Phenochart v1.0.8 image software (Akoya Biosciences). Supplementary Table 4 summarizes the number of slides included in this study.

#### Detection of SARS-CoV-2 viral RNA in formalin-fixed paraffin-embedded (FFPE) placental tissues

From each patient [7 SARS-CoV-2 (+) and 3 healthy pregnant women], 6–14 sections of 10-μm-thick FFPE basal plate, placental villi, and the chorioamniotic membranes were used for total RNA isolation using the PureLink™ FFPE Total RNA Isolation Kit (Invitrogen), according to the manufacturer’s instructions. Samples of the basal plate, placental villi, and chorioamniotic membranes were spiked with heat inactivated SARS-Related Coronavirus 2 (SARS-CoV-2) Isolate USA-WA 1/2020 as a positive control prior to formalin fixation and paraffin embedding. Total RNA was isolated from spiked tissues as described above. Following the instructions from the CDC-2019 Novel Coronavirus (2019-nCoV) Real-Time RT-PCR Diagnostic Panel, cDNA was synthesized using TaqPath™ 1-Step RT-qPCR Master Mix, CG and primers from the 2019-nCoV RUO Kit. Reactions were incubated at 25°C for 2 min followed by 50°C for 15 min. Initial denaturation was set for 2 min at 95°C followed by 45 amplification cycles at 95°C for 3 sec and 55°C for 30 sec. A cycle of quantification (C_q_ value) less than 45 indicates a positive result. Two positive PCR controls were used: 2019-nCoV_N (virus) and Hs_RPP30 (human). Each PCR sample was run in duplicate.

### Molecular microbiology

#### Sample collection

Swabs (FLOQSwabs; COPAN, Murrieta, CA, USA) for molecular microbiology were collected from the chorioamniotic membranes, the amnion-chorion interface of the placental disc, and the placental villous tree. These swabs were stored at −80°C until DNA extractions were performed.

#### DNA extraction

All DNA extractions were completed within a biological safety cabinet using a DNeasy Powerlyzer Powersoil Kit (Qiagen, Germantown, MD, USA), with minor modifications to the manufacturer’s instructions as previously described^112,114^. Personnel wore sterile surgical masks, gowns, and gloves during the procedure. Briefly, following UV treatment, 400 μL of Powerbead solution, 200 μL of phenol:chloroform:isoamyl alcohol (pH 7–8), and 60 μL of pre-heated solution C1 were added to the bead tubes. The swab samples were added to the tubes, incubated for 10 minutes, and then mechanically lysed for two rounds of 30 sec each using a bead beater. Following a 1 min centrifugation and transferring of the supernatant to new tubes, 100 μL of PureLink™ RNase A (20 mg/mL) (Invitrogen), 100 μL of solution C2, and 100 μL of solution C3 were added. The tubes were incubated at 4°C for 5 min and centrifuged for 1 min. After transferring the lysates to new tubes, 650 μL of solution C4 and 650 μL of 100% ethanol were added. Next, 635 μL of the lysate were loaded onto the filter columns and centrifuged for 1 min, discarding the flowthrough. This wash step was repeated three times to ensure all the lysates passed through the columns. Following the washes, 500 μL of solution C5 were added to the filter columns. After a 1 min centrifugation, the flowthrough was discarded and the tubes were centrifuged again for 2 min to dry the spin columns. The spin columns themselves were transferred to a clean 2.0 mL collection tube, and 60 μL of pre-heated solution C6 was added directly to the center of the spin columns. After a 5 min incubation at room temperature, the DNA was eluted via a 1 min centrifugation. Purified DNA was then transferred to clean 2.0 mL collection tubes and immediately stored at −20°C. Twelve extractions of sterile FLOQSwabs were included as technical controls for potential background DNA contamination.

#### 16s rRNA gene quantitative real-time PCR

Total bacterial DNA abundance within samples was measured via amplification of the V1 - V2 region of the 16S rRNA gene according to the protocol of Dickson et al.^140^ with minor modifications, as previously described^112,114^. These modifications included the use of a degenerative forward primer (27f-CM: 5’-AGA GTT TGA TCM TGG CTC AG-3’) and a degenerate probe containing locked nucleic acids (+) (BSR65/17: 5’−56FAM-TAA +YA+C ATG +CA+A GT+C GA-BHQ1–3’). Each 20 μL reaction contained 0.6 μM of 27f-CM primer, 0.6 μM of 357R primer (5’-CTG CTG CCT YCC GTA G-3’), 0.25 μM of BSR65/17 probe, 10.0 μL of 2X TaqMan Environmental Master Mix 2.0 (Invitrogen), and 3.0 μL of either purified DNA or nuclease-free water. The total bacterial DNA qPCR was performed using the following conditions: 95°C for 10 min, followed by 40 cycles of 94°C for 30 sec, 50°C for 30 sec, and 72°C for 30 sec. Duplicate reactions were run for all samples.

Raw amplification data were normalized to the ROX passive reference dye and analyzed using the 7500 Software version 2.3 (Applied Biosystems, Foster City, CA, USA) with automatic threshold and baseline settings. Cycle of quantification (Cq) values were calculated for samples based on the mean number of cycles required for normalized fluorescence to exponentially increase.

#### 16S rRNA gene sequencing and processing

Amplification and sequencing of the V4 region of the 16S rRNA gene was performed using the dual indexing sequencing strategy developed by Kozich et al.^141^. The forward primer was 515F: 5’-GTGCCAGCMGCCGCGGTAA-3’ and the reverse primer was 806R: 5’-GGACTACHVGGGTWTCTAAT-3’. Each PCR reaction contained 0.75 nM of each primer, 3.0 μL template DNA, 10.0 μL of 2X TaqMan Environmental Master Mix 2.0, and DNase-free water to produce a final volume of 20 μL. Reactions were performed using the following conditions: 95°C for 10 min, followed by 40 cycles of 95°C for 20 sec, 55°C for 15 sec, and 72°C for 5 min, with an additional elongation at 72°C for 10 min. All PCR reactions were run in duplicate and products from duplicate reactions were pooled prior to purification and sequencing.

16S rRNA gene sequencing libraries were prepared according to Illumina’s protocol for Preparing Libraries for Sequencing on the MiSeq (15039740 Rev. D) for 2 nM or 4 nM libraries. Sequencing was conducted using the Illumina MiSeq platform (V2 500 cycles, Illumina MS102–2003), according to the manufacturer’s instructions with modifications found in ^141^. All samples were quantified using the Qubit dsDNA HS assay and pooled in equimolar concentration prior to sequencing.

16S rRNA gene sequences were clustered into amplicon sequence variants (ASVs) defined by 100% sequence similarity using DADA2 version 1.12^142^ in R version 3.6.1^143^ according to the online MiSeq protocol (https://benjjneb.github.io/dada2/tutorial.html) with minor modifications, as previously described^114^. These modifications included allowing truncation lengths of 250 and 150 bases, and a maximum number of expected errors of 2 and 7 bases, for forward and reverse reads, respectively. To increase power for detecting rare variants, sample inference allowed for pooling of samples. Additionally, samples in the resulting sequence table were pooled prior to removal of chimeric sequences. Sequences were then classified using the silva_nr_v132_train_set database with a minimum bootstrap value of 80%, and sequences that were derived from Archaea, chloroplast, or Eukaryota were removed.

The R package decontam version 1.6.0^144^ was used to identify ASVs that were potential background DNA contaminants based on their pattern of occurrence in biological versus technical control samples using the “IsNotContaminant” function. An ASV was determined to be a contaminant, and was thus removed from the entire dataset, if it had a P score ≥ 0.4, had a higher mean relative abundance in technical controls than biological samples, and was present in more than one-third of technical control samples. Although one ASV, which was classified as *Lactobacillus*, met all the criteria for being defined as a contaminant, it was highly abundant in all three positive control vaginal samples and was therefore not removed from the dataset. Ultimately, a total of 148 ASVs were determined to be contaminants and were removed from the dataset prior to analysis. The vast majority of these ASVs were classified as *Staphylococcus* (138/148 ASVs; 93.2%).

#### 16S rRNA gene profile statistical analyses

Prior to analyses, the dataset was randomly subsampled to 5,426 sequences per sample. Heatmaps of the 16S rRNA gene profiles of samples, including all prominent ASVs (i.e. those ASVs with an average relative abundance ≥ 2% for any placental site and/or mode of delivery combination) were generated using the open-source software program Morpheus (https://software.broadinstitute.org/morpheus). Differences in the structure of 16S rRNA gene profiles of samples were assessed using the Bray-Curtis dissimilarity index. Variation in the 16S rRNA gene profiles of the placental samples from different study groups were visualized through Principal Coordinates Analyses (PCoA) using the R package vegan version 2.5–6^145^. Statistical evaluation of 16S rRNA gene profile differences between study groups was completed using permutational multivariate analysis of variance (PERMANOVA)^146^ through the “adonis” function in the R package vegan version 2.5–6.

### Statistical analysis

Statistical analyses were performed using SPSS v19.0 (IBM, Armonk, NY, USA) or the R package (as described above). For human demographic data, the group comparisons were performed using the Fisher’s exact test for proportions and Mann-Whitney U-test for non-normally distributed continuous variables. Immunoglobulin and cytokine/chemokine concentrations were compared using Mann-Whitney U-tests. Principal component analysis (PCA) of cytokines detected in all samples was performed using the R package PCAtools after separately normalizing the data from maternal and cord blood. A two-sample student’s t-test was used to assess whether the first principal component (PC1) values were different between SARS-CoV-2-infected and control groups. For the comparison of flow cytometry data between study groups, Mann-Whitney U-tests were also performed. P < 0.05 was considered statistically significant. For heatmap representation of immunophenotyping results, flow cytometry data were transformed into Z-scores by subtracting the mean and dividing by the standard deviation, which were both calculated from the control group. The Z-scores were visualized as a heat map and compared between SARS-CoV-2 (+) and control groups using two-sample t-tests. P-values were adjusted for multiple comparisons using the false discovery rate method to obtain q-values. A q-value < 0.1 was considered statistically significant. The principal components (PC) of the flow cytometry data were also determined, and PC1, PC2, and PC3 were plotted on a 3D scatter plot. Single-cell RNAseq and MiSeq data analyses were performed as described in their respective sections.

## DATA AVAILABILITY

The majority of the data generated in this study are included in the manuscript or in the Supplementary Materials.

The genotyping and single-cell RNAseq data reported in this study have been submitted to the NIH dbGAP repository (accession number phs001886.v3.p1). All software and R packages used herein are detailed in the Materials and Methods. Scripts detailing the single-cell analyses are also available at https://github.com/piquelab/covid19placenta.

The raw MiSeq data reported in this study have been deposited in the NCBI Sequence Read Archive (Bioproject ID: PRJNA701628).

## Supplementary Material

Supplement

Supplement

## References

[1] CDC. Data on COVID-19 during Pregnancy, <https://www.cdc.gov/coronavirus/2019-ncov/cases-updates/special-populations/pregnancy-data-on-covid-19.html≥ (2021).

[2] Panagiotakopoulos L. SARS-CoV-2 Infection Among Hospitalized Pregnant Women: Reasons for Admission and Pregnancy Characteristics - Eight U.S. Health Care Centers, March 1-May 30, 2020. MMWR Morb Mortal Wkly Rep 69, 1355–1359, doi:10.15585/mmwr.mm6938e2 (2020). 32970660 PMC7727498

[3] Lokken E. M. Clinical characteristics of 46 pregnant women with a severe acute respiratory syndrome coronavirus 2 infection in Washington State. Am J Obstet Gynecol 223, 911.e911–911.e914, doi:10.1016/j.ajog.2020.05.031 (2020). 32439389 PMC7234933

[4] Sutton D., Fuchs K., D’Alton M. & Goffman D. Universal Screening for SARS-CoV-2 in Women Admitted for Delivery. N Engl J Med, doi:10.1056/NEJMc2009316 (2020). PMC7175422 32283004

[5] Chen L. Clinical Characteristics of Pregnant Women with Covid-19 in Wuhan, China. N Engl J Med, doi:10.1056/NEJMc2009226 (2020). PMC7182016 32302077

[6] Ellington S. Characteristics of Women of Reproductive Age with Laboratory-Confirmed SARS-CoV-2 Infection by Pregnancy Status - United States, January 22-June 7, 2020. MMWR Morb Mortal Wkly Rep 69, 769–775, doi:10.15585/mmwr.mm6925a1 (2020). 32584795 PMC7316319

[7] Pierce-Williams R. A. M. Clinical course of severe and critical coronavirus disease 2019 in hospitalized pregnancies: a United States cohort study. Am J Obstet Gynecol MFM 2, 100134, doi:10.1016/j.ajogmf.2020.100134 (2020). 32391519 PMC7205698

[8] Woodworth K. R. Birth and Infant Outcomes Following Laboratory-Confirmed SARS-CoV-2 Infection in Pregnancy - SET-NET, 16 Jurisdictions, March 29-October 14, 2020. MMWR Morb Mortal Wkly Rep 69, 1635–1640, doi:10.15585/mmwr.mm6944e2 (2020). 33151917 PMC7643898

[9] Zambrano L. D. Update: Characteristics of Symptomatic Women of Reproductive Age with Laboratory-Confirmed SARS-CoV-2 Infection by Pregnancy Status - United States, January 22-October 3, 2020. MMWR Morb Mortal Wkly Rep 69, 1641–1647, doi:10.15585/mmwr.mm6944e3 (2020). 33151921 PMC7643892

[10] CDC. Investigating the Impact of COVID-19 during Pregnancy, <https://www.cdc.gov/coronavirus/2019-ncov/cases-updates/special-populations/pregnancy-data-on-covid-19/what-cdc-is-doing.html≥ (2021).

[11] Lokken E. M. Disease Severity, Pregnancy Outcomes and Maternal Deaths among Pregnant Patients with SARS-CoV-2 Infection in Washington State. Am J Obstet Gynecol, doi:10.1016/j.ajog.2020.12.1221 (2021). PMC7838012 33515516

[12] Lokken E. M. Higher SARS-CoV-2 Infection Rate in Pregnant Patients. Am J Obstet Gynecol, doi:10.1016/j.ajog.2021.02.011 (2021). PMC7884918 33607103

[13] Raschetti R. Synthesis and systematic review of reported neonatal SARS-CoV-2 infections. Nat Commun 11, 5164, doi:10.1038/s41467-020-18982-9 (2020). 33060565 PMC7566441

[14] World Health O. Definition and categorization of the timing of mother-to-child transmission of SARS-CoV-2: scientific brief, 8 2 2021. (World Health Organization, Geneva, 2021).

[15] Vivanti A. J. Transplacental transmission of SARS-CoV-2 infection. Nat Commun 11, 3572, doi:10.1038/s41467-020-17436-6 (2020). 32665677 PMC7360599

[16] Fenizia C. Analysis of SARS-CoV-2 vertical transmission during pregnancy. Nat Commun 11, 5128, doi:10.1038/s41467-020-18933-4 (2020). 33046695 PMC7552412

[17] Schwartz D. A. & Dhaliwal A. Infections in pregnancy with COVID-19 and other respiratory RNA virus diseases are rarely, if ever, transmitted to the fetus: Experiences with coronaviruses, HPIV, HMPV RSV, and influenza. Arch Pathol Lab Med, doi:10.5858/arpa.2020-0211-SA (2020). 32338533

[18] Simmons G., Zmora P., Gierer S., Heurich A. & Pöhlmann S. Proteolytic activation of the SARS-coronavirus spike protein: cutting enzymes at the cutting edge of antiviral research. Antiviral Res 100, 605–614, doi:10.1016/j.antiviral.2013.09.028 (2013). 24121034 PMC3889862

[19] Phillips J. M., Gallagher T. & Weiss S. R. Neurovirulent Murine Coronavirus JHM.SD Uses Cellular Zinc Metalloproteases for Virus Entry and Cell-Cell Fusion. J Virol 91, doi:10.1128/jvi.01564-16 (2017). PMC5375694 28148786

[20] Shang J. Structural basis of receptor recognition by SARS-CoV-2. Nature, doi:10.1038/s41586-020-2179-y (2020). PMC7328981 32225175

[21] Hoffmann M. SARS-CoV-2 Cell Entry Depends on ACE2 and TMPRSS2 and Is Blocked by a Clinically Proven Protease Inhibitor. Cell 181, 271–280 e278, doi:10.1016/j.cell.2020.02.052 (2020). 32142651 PMC7102627

[22] Pique-Regi R. Does the human placenta express the canonical cell entry mediators for SARS-CoV-2? Elife 9, doi:10.7554/eLife.58716 (2020). PMC7367681 32662421

[23] Hecht J. L. SARS-CoV-2 can infect the placenta and is not associated with specific placental histopathology: a series of 19 placentas from COVID-19-positive mothers. Mod Pathol 33, 2092–2103, doi:10.1038/s41379-020-0639-4 (2020). 32741970 PMC7395938

[24] Edlow A. G. Assessment of Maternal and Neonatal SARS-CoV-2 Viral Load, Transplacental Antibody Transfer, and Placental Pathology in Pregnancies During the COVID-19 Pandemic. JAMA Netw Open 3, e2030455, doi:10.1001/jamanetworkopen.2020.30455 (2020). 33351086 PMC7756241

[25] Sharps M. C. A structured review of placental morphology and histopathological lesions associated with SARS-CoV-2 infection. Placenta 101, 13–29, doi:10.1016/j.placenta.2020.08.018 (2020). 32911234 PMC7443324

[26] Patanè L. Vertical transmission of coronavirus disease 2019: severe acute respiratory syndrome coronavirus 2 RNA on the fetal side of the placenta in pregnancies with coronavirus disease 2019-positive mothers and neonates at birth. Am J Obstet Gynecol MFM 2, 100145, doi:10.1016/j.ajogmf.2020.100145 (2020). 32427221 PMC7233206

[27] Hosier H. SARS-CoV-2 infection of the placenta. J Clin Invest 130, 4947–4953, doi:10.1172/jci139569 (2020). 32573498 PMC7456249

[28] Algarroba G. N. Visualization of severe acute respiratory syndrome coronavirus 2 invading the human placenta using electron microscopy. Am J Obstet Gynecol 223, 275–278, doi:10.1016/j.ajog.2020.05.023 (2020). 32405074 PMC7219376

[29] Flannery D. D. Assessment of Maternal and Neonatal Cord Blood SARS-CoV-2 Antibodies and Placental Transfer Ratios. JAMA Pediatr, doi:10.1001/jamapediatrics.2021.0038 (2021). PMC7846944 33512440

[30] Dong L. Possible Vertical Transmission of SARS-CoV-2 From an Infected Mother to Her Newborn. JAMA, doi:10.1001/jama.2020.4621 (2020). PMC7099527 32215581

[31] Tay M. Z., Poh C. M., Renia L., MacAry P. A. & Ng L. F. P. The trinity of COVID-19: immunity, inflammation and intervention. Nat Rev Immunol 20, 363–374, doi:10.1038/s41577-020-0311-8 (2020). 32346093 PMC7187672

[32] Lucas C. Longitudinal analyses reveal immunological misfiring in severe COVID-19. Nature 584, 463–469, doi:10.1038/s41586-020-2588-y (2020). 32717743 PMC7477538

[33] Song J. W. Immunological and inflammatory profiles in mild and severe cases of COVID-19. Nat Commun 11, 3410, doi:10.1038/s41467-020-17240-2 (2020). 32641700 PMC7343781

[34] Bernardes J. P. Longitudinal Multi-omics Analyses Identify Responses of Megakaryocytes, Erythroid Cells, and Plasmablasts as Hallmarks of Severe COVID-19. Immunity 53, 1296–1314 e1299, doi:10.1016/j.immuni.2020.11.017 (2020). 33296687 PMC7689306

[35] Schulte-Schrepping J. Severe COVID-19 Is Marked by a Dysregulated Myeloid Cell Compartment. Cell 182, 1419–1440 e1423, doi:10.1016/j.cell.2020.08.001 (2020). 32810438 PMC7405822

[36] Hadjadj J. Impaired type I interferon activity and inflammatory responses in severe COVID-19 patients. Science 369, 718–724, doi:10.1126/science.abc6027 (2020). 32661059 PMC7402632

[37] Burton G. J. & Jauniaux E. What is the placenta? Am J Obstet Gynecol 213, S6 e1, S6–8, doi:10.1016/j.ajog.2015.07.050 (2015). 26428504

[38] Maltepe E. & Fisher S. J. Placenta: the forgotten organ. Annu Rev Cell Dev Biol 31, 523–552, doi:10.1146/annurev-cellbio-100814-125620 (2015). 26443191

[39] Pique-Regi R. Single cell transcriptional signatures of the human placenta in term and preterm parturition. Elife 8, doi:10.7554/eLife.52004 (2019). PMC6949028 31829938

[40] Meckiff B. J. Imbalance of Regulatory and Cytotoxic SARS-CoV-2-Reactive CD4(+) T Cells in COVID-19. Cell 183, 1340–1353 e1316, doi:10.1016/j.cell.2020.10.001 (2020). 33096020 PMC7534589

[41] Bost P. Host-Viral Infection Maps Reveal Signatures of Severe COVID-19 Patients. Cell 181, 1475–1488 e1412, doi:10.1016/j.cell.2020.05.006 (2020). 32479746 PMC7205692

[42] Egerup P. Severe Acute Respiratory Syndrome Coronavirus 2 (SARS-CoV-2) Antibodies at Delivery in Women, Partners, and Newborns. Obstet Gynecol 137, 49–55, doi:10.1097/AOG.0000000000004199 (2021). 33116054

[43] Crovetto F. Impact of SARS-CoV-2 Infection on Pregnancy Outcomes: A Population-Based Study. Clin Infect Dis, doi:10.1093/cid/ciab104 (2021). PMC7929066 33556958

[44] Simister N. E., Story C. M., Chen H. L. & Hunt J. S. An IgG-transporting Fc receptor expressed in the syncytiotrophoblast of human placenta. Eur J Immunol 26, 1527–1531, doi:10.1002/eji.1830260718 (1996). 8766556

[45] Leach J. L. Isolation from human placenta of the IgG transporter, FcRn, and localization to the syncytiotrophoblast: implications for maternal-fetal antibody transport. J Immunol 157, 3317–3322 (1996). 8871627

[46] Atyeo C. Compromised SARS-CoV-2-specific placental antibody transfer. Cell 184, 628–642 e610, doi:10.1016/j.cell.2020.12.027 (2021). 33476549 PMC7755577

[47] Haider S. A. Serum IgM in diagnosis of infection in the newborn. Arch Dis Child 47, 382–393, doi:10.1136/adc.47.253.382 (1972). 4556162 PMC1648108

[48] Guerina N. G. Neonatal serologic screening and early treatment for congenital Toxoplasma gondii infection. The New England Regional Toxoplasma Working Group. N Engl J Med 330, 1858–1863, doi:10.1056/NEJM199406303302604 (1994). 7818637

[49] Long Q. X. Clinical and immunological assessment of asymptomatic SARS-CoV-2 infections. Nat Med 26, 1200–1204, doi:10.1038/s41591-020-0965-6 (2020). 32555424

[50] Miossec P., Korn T. & Kuchroo V. K. Interleukin-17 and type 17 helper T cells. N Engl J Med 361, 888–898, doi:10.1056/NEJMra0707449 (2009). 19710487

[51] Liu Y. 2019-novel coronavirus (2019-nCoV) infections trigger an exaggerated cytokine response aggravating lung injury. ChinaXiv chinaXiv:202002.00018V1 (2020).

[52] Xu Z. Pathological findings of COVID-19 associated with acute respiratory distress syndrome. Lancet Respir Med 8, 420–422, doi:10.1016/S2213-2600(20)30076-X (2020). 32085846 PMC7164771

[53] Huang C. Clinical features of patients infected with 2019 novel coronavirus in Wuhan, China. Lancet 395, 497–506, doi:10.1016/S0140-6736(20)30183-5 (2020). 31986264 PMC7159299

[54] Pacha O., Sallman M. A. & Evans S. E. COVID-19: a case for inhibiting IL-17? Nat Rev Immunol 20, 345–346, doi:10.1038/s41577-020-0328-z (2020). 32358580 PMC7194244

[55] Aggarwal B. B. Signalling pathways of the TNF superfamily: a double-edged sword. Nat Rev Immunol 3, 745–756, doi:10.1038/nri1184 (2003). 12949498

[56] Diao B. Reduction and Functional Exhaustion of T Cells in Patients With Coronavirus Disease 2019 (COVID-19). Front Immunol 11, 827, doi:10.3389/fimmu.2020.00827 (2020). 32425950 PMC7205903

[57] Zaretsky M. V., Alexander J. M., Byrd W. & Bawdon R. E. Transfer of inflammatory cytokines across the placenta. Obstet Gynecol 103, 546–550, doi:10.1097/01.AOG.0000114980.40445.83 (2004). 14990420

[58] Dahlgren J., Samuelsson A. M., Jansson T. & Holmang A. Interleukin-6 in the maternal circulation reaches the rat fetus in mid-gestation. Pediatr Res 60, 147–151, doi:10.1203/01.pdr.0000230026.74139.18 (2006). 16864694

[59] Tanaka T., Narazaki M. & Kishimoto T. IL-6 in inflammation, immunity, and disease. Cold Spring Harb Perspect Biol 6, a016295, doi:10.1101/cshperspect.a016295 (2014). 25190079 PMC4176007

[60] Gubernatorova E. O., Gorshkova E. A., Polinova A. I. & Drutskaya M. S. IL-6: Relevance for immunopathology of SARS-CoV-2. Cytokine Growth Factor Rev 53, 13–24, doi:10.1016/j.cytogfr.2020.05.009 (2020). 32475759 PMC7237916

[61] Prins J. R., Gomez-Lopez N. & Robertson S. A. Interleukin-6 in pregnancy and gestational disorders. J Reprod Immunol 95, 1–14, doi:10.1016/j.jri.2012.05.004 (2012). 22819759

[62] Liu T. The role of interleukin-6 in monitoring severe case of coronavirus disease 2019. EMBO Mol Med 12, e12421, doi:10.15252/emmm.202012421 (2020). 32428990 PMC7280589

[63] Zhang J. Serum interleukin-6 is an indicator for severity in 901 patients with SARS-CoV-2 infection: a cohort study. J Transl Med 18, 406, doi:10.1186/s12967-020-02571-x (2020). 33121497 PMC7594951

[64] Tavakolpour S., Rakhshandehroo T., Wei E. X. & Rashidian M. Lymphopenia during the COVID-19 infection: What it shows and what can be learned. Immunol Lett 225, 31–32, doi:10.1016/j.imlet.2020.06.013 (2020). 32569607 PMC7305732

[65] Jiang M. T-Cell Subset Counts in Peripheral Blood Can Be Used as Discriminatory Biomarkers for Diagnosis and Severity Prediction of Coronavirus Disease 2019. J Infect Dis 222, 198–202, doi:10.1093/infdis/jiaa252 (2020). 32379887 PMC7239156

[66] Schub D. High levels of SARS-CoV-2-specific T cells with restricted functionality in severe courses of COVID-19. JCI Insight 5, doi:10.1172/jci.insight.142167 (2020). PMC7605520 32937615

[67] Chen Z. & John Wherry E. T cell responses in patients with COVID-19. Nat Rev Immunol 20, 529–536, doi:10.1038/s41577-020-0402-6 (2020). 32728222 PMC7389156

[68] Giamarellos-Bourboulis E. J. Complex Immune Dysregulation in COVID-19 Patients with Severe Respiratory Failure. Cell Host Microbe 27, 992–1000 e1003, doi:10.1016/j.chom.2020.04.009 (2020). 32320677 PMC7172841

[69] Liu Z. Lymphocyte subset (CD4+, CD8+) counts reflect the severity of infection and predict the clinical outcomes in patients with COVID-19. J Infect 81, 318–356, doi:10.1016/j.jinf.2020.03.054 (2020). PMC7151318 32283159

[70] De Biasi S. Marked T cell activation, senescence, exhaustion and skewing towards TH17 in patients with COVID-19 pneumonia. Nat Commun 11, 3434, doi:10.1038/s41467-020-17292-4 (2020). 32632085 PMC7338513

[71] Mathew D. Deep immune profiling of COVID-19 patients reveals distinct immunotypes with therapeutic implications. Science 369, doi:10.1126/science.abc8511 (2020). PMC7402624 32669297

[72] Zhang X. Viral and host factors related to the clinical outcome of COVID-19. Nature 583, 437–440, doi:10.1038/s41586-020-2355-0 (2020). 32434211

[73] Yang H. Clinical features and outcomes of pregnant women suspected of coronavirus disease 2019. J Infect 81, e40–e44, doi:10.1016/j.jinf.2020.04.003 (2020). 32294503 PMC7152867

[74] Andrikopoulou M. Symptoms and Critical Illness Among Obstetric Patients With Coronavirus Disease 2019 (COVID-19) Infection. Obstet Gynecol 136, 291–299, doi:10.1097/AOG.0000000000003996 (2020). 32459701

[75] Emmi L. & Romagnami S. in The Autoimmune Diseases (Fourth Edition) (eds Rose Noel R. & Mackay Ian R.) 83–101 (Academic Press, 2006).

[76] Hamada H. Tc17, a unique subset of CD8 T cells that can protect against lethal influenza challenge. J Immunol 182, 3469–3481, doi:10.4049/jimmunol.0801814 (2009). 19265125 PMC2667713

[77] Saito S., Nakashima A., Shima T. & Ito M. Th1/Th2/Th17 and regulatory T-cell paradigm in pregnancy. Am J Reprod Immunol 63, 601–610, doi:10.1111/j.1600-0897.2010.00852.x (2010). 20455873

[78] Gomez-Lopez N. Regulatory T Cells Play a Role in a Subset of Idiopathic Preterm Labor/Birth and Adverse Neonatal Outcomes. Cell Rep 32, 107874, doi:10.1016/j.celrep.2020.107874 (2020). 32640239 PMC7396155

[79] Miller D., Gershater M., Slutsky R., Romero R. & Gomez-Lopez N. Maternal and fetal T cells in term pregnancy and preterm labor. Cell Mol Immunol 17, 693–704, doi:10.1038/s41423-020-0471-2 (2020). 32467619 PMC7331691

[80] Chaouat G., Voisin G. A., Escalier D. & Robert P. Facilitation reaction (enhancing antibodies and suppressor cells) and rejection reaction (sensitized cells) from the mother to the paternal antigens of the conceptus. Clin Exp Immunol 35, 13–24 (1979). 371881 PMC1537606

[81] Aluvihare V. R., Kallikourdis M. & Betz A. G. Regulatory T cells mediate maternal tolerance to the fetus. Nat Immunol 5, 266–271, doi:10.1038/ni1037 (2004). 14758358

[82] Zenclussen A. C. Abnormal T-cell reactivity against paternal antigens in spontaneous abortion: adoptive transfer of pregnancy-induced CD4+CD25+ T regulatory cells prevents fetal rejection in a murine abortion model. Am J Pathol 166, 811–822, doi:10.1016/s0002-9440(10)62302-4 (2005). 15743793 PMC1602357

[83] Robertson S. A., Guerin L. R., Moldenhauer L. M. & Hayball J. D. Activating T regulatory cells for tolerance in early pregnancy - the contribution of seminal fluid. J Reprod Immunol 83, 109–116, doi:10.1016/j.jri.2009.08.003 (2009). 19875178

[84] Kahn D. A. & Baltimore D. Pregnancy induces a fetal antigen-specific maternal T regulatory cell response that contributes to tolerance. Proc Natl Acad Sci U S A 107, 9299–9304, doi:10.1073/pnas.1003909107 (2010). 20439708 PMC2889122

[85] Shima T. Regulatory T cells are necessary for implantation and maintenance of early pregnancy but not late pregnancy in allogeneic mice. J Reprod Immunol 85, 121–129, doi:10.1016/j.jri.2010.02.006 (2010). 20439117

[86] Samstein R. M., Josefowicz S. Z., Arvey A., Treuting P. M. & Rudensky A. Y. Extrathymic generation of regulatory T cells in placental mammals mitigates maternal-fetal conflict. Cell 150, 29–38, doi:10.1016/j.cell.2012.05.031 (2012). 22770213 PMC3422629

[87] Rowe J. H., Ertelt J. M., Xin L. & Way S. S. Pregnancy imprints regulatory memory that sustains anergy to fetal antigen. Nature 490, 102–106, doi:10.1038/nature11462 (2012). 23023128 PMC3465465

[88] Matthiesen L., Kalkunte S. & Sharma S. Multiple pregnancy failures: an immunological paradigm. Am J Reprod Immunol 67, 334–340, doi:10.1111/j.1600-0897.2012.01121.x (2012). 22380628

[89] PrabhuDas M. Immune mechanisms at the maternal-fetal interface: perspectives and challenges. Nat Immunol 16, 328–334, doi:10.1038/ni.3131 (2015). 25789673 PMC5070970

[90] Bonney E. A. Alternative theories: Pregnancy and immune tolerance. J Reprod Immunol 123, 65–71, doi:10.1016/j.jri.2017.09.005 (2017). 28941880

[91] Gomez-Lopez N., Guilbert L. J. & Olson D. M. Invasion of the leukocytes into the fetal-maternal interface during pregnancy. J Leukoc Biol 88, 625–633, doi:10.1189/jlb.1209796 (2010). 20519637

[92] Arenas-Hernandez M. Effector and Activated T Cells Induce Preterm Labor and Birth That Is Prevented by Treatment with Progesterone. J Immunol 202, 2585–2608, doi:10.4049/jimmunol.1801350 (2019). 30918041 PMC6570421

[93] Slutsky R. Exhausted and Senescent T Cells at the Maternal-Fetal Interface in Preterm and Term Labor. J Immunol Res 2019, 3128010, doi:10.1155/2019/3128010 (2019). 31263712 PMC6556261

[94] Gomez-Lopez N. Evidence for a role for the adaptive immune response in human term parturition. Am J Reprod Immunol 69, 212–230, doi:10.1111/aji.12074 (2013). 23347265 PMC3600361

[95] Tarca A. L. Targeted expression profiling by RNA-Seq improves detection of cellular dynamics during pregnancy and identifies a role for T cells in term parturition. Sci Rep 9, 848, doi:10.1038/s41598-018-36649-w (2019). 30696862 PMC6351599

[96] Almeida L. Ribosome-Targeting Antibiotics Impair T Cell Effector Function and Ameliorate Autoimmunity by Blocking Mitochondrial Protein Synthesis. Immunity 54, 68–83 e66, doi:10.1016/j.immuni.2020.11.001 (2021). 33238133 PMC7837214

[97] Chua R. L. COVID-19 severity correlates with airway epithelium-immune cell interactions identified by single-cell analysis. Nat Biotechnol 38, 970–979, doi:10.1038/s41587-020-0602-4 (2020). 32591762

[98] Liao M. Single-cell landscape of bronchoalveolar immune cells in patients with COVID-19. Nat Med 26, 842–844, doi:10.1038/s41591-020-0901-9 (2020). 32398875

[99] Merad M. & Martin J. C. Pathological inflammation in patients with COVID-19: a key role for monocytes and macrophages. Nat Rev Immunol 20, 355–362, doi:10.1038/s41577-020-0331-4 (2020). 32376901 PMC7201395

[100] Yu N. Clinical features and obstetric and neonatal outcomes of pregnant patients with COVID-19 in Wuhan, China: a retrospective, single-centre, descriptive study. Lancet Infect Dis 20, 559–564, doi:10.1016/S1473-3099(20)30176-6 (2020). 32220284 PMC7158904

[101] Levitan D. Histologic and Immunohistochemical Evaluation of 65 Placentas from Women with Polymerase Chain Reaction-proven Severe Acute Respiratory Syndrome Coronavirus 2 (SARS-CoV-2) Infection. Arch Pathol Lab Med, doi:10.5858/arpa.2020-0793-SA (2021). 33596304

[102] Chen H. Clinical characteristics and intrauterine vertical transmission potential of COVID-19 infection in nine pregnant women: a retrospective review of medical records. Lancet 395, 809–815, doi:10.1016/S0140-6736(20)30360-3 (2020). 32151335 PMC7159281

[103] Yan J. Coronavirus disease 2019 in pregnant women: a report based on 116 cases. Am J Obstet Gynecol 223, 111 e111–111 e114, doi:10.1016/j.ajog.2020.04.014 (2020). 32335053 PMC7177142

[104] Yu N. No SARS-CoV-2 detected in amniotic fluid in mid-pregnancy. Lancet Infect Dis 20, 1364, doi:10.1016/S1473-3099(20)30320-0 (2020). 32333848 PMC7176395

[105] Cribiu F. M. Severe SARS-CoV-2 placenta infection can impact neonatal outcome in the absence of vertical transmission. J Clin Invest, doi:10.1172/JCI145427 (2021). PMC7954587 33497369

[106] Kotlyar A. M. Vertical transmission of coronavirus disease 2019: a systematic review and meta-analysis. Am J Obstet Gynecol 224, 35–53 e33, doi:10.1016/j.ajog.2020.07.049 (2021). 32739398 PMC7392880

[107] Funkhouser L. J. & Bordenstein S. R. Mom knows best: the universality of maternal microbial transmission. PLoS Biol 11, e1001631, doi:10.1371/journal.pbio.1001631 (2013). 23976878 PMC3747981

[108] Perez-Munoz M. E., Arrieta M. C., Ramer-Tait A. E. & Walter J. A critical assessment of the “sterile womb” and “in utero colonization” hypotheses: implications for research on the pioneer infant microbiome. Microbiome 5, 48, doi:10.1186/s40168-017-0268-4 (2017). 28454555 PMC5410102

[109] Theis K. R. Does the human placenta delivered at term have a microbiota? Results of cultivation, quantitative real-time PCR, 16S rRNA gene sequencing, and metagenomics. Am J Obstet Gynecol 220, 267 e261–267 e239, doi:10.1016/j.ajog.2018.10.018 (2019). 30832984 PMC6733039

[110] de Goffau M. C. Human placenta has no microbiome but can contain potential pathogens. Nature 572, 329–334, doi:10.1038/s41586-019-1451-5 (2019). 31367035 PMC6697540

[111] Sterpu I. No evidence for a placental microbiome in human pregnancies at term. Am J Obstet Gynecol, doi:10.1016/j.ajog.2020.08.103 (2020). 32871131

[112] Theis K. R. No Consistent Evidence for Microbiota in Murine Placental and Fetal Tissues. mSphere 5, doi:10.1128/mSphere.00933-19 (2020). PMC7045391 32102944

[113] Kuperman A. A. Deep microbial analysis of multiple placentas shows no evidence for a placental microbiome. BJOG 127, 159–169, doi:10.1111/1471-0528.15896 (2020). 31376240

[114] Theis K. R., Romero R., Winters A. D., Jobe A. H. & Gomez-Lopez N. Lack of Evidence for Microbiota in the Placental and Fetal Tissues of Rhesus Macaques. mSphere 5, doi:10.1128/mSphere.00210-20 (2020). PMC7203455 32376701

[115] ACOG. Gestational Hypertension and Preeclampsia: ACOG Practice Bulletin, Number 222. Obstet Gynecol 135, e237–e260, doi:10.1097/AOG.0000000000003891 (2020). 32443079

[116] Khong T. Y. Sampling and Definitions of Placental Lesions: Amsterdam Placental Workshop Group Consensus Statement. Arch Pathol Lab Med 140, 698–713, doi:10.5858/arpa.2015-0225-CC (2016). 27223167

[117] Kim C. J. Acute chorioamnionitis and funisitis: definition, pathologic features, and clinical significance. Am J Obstet Gynecol 213, S29–52, doi:10.1016/j.ajog.2015.08.040 (2015). 26428501 PMC4774647

[118] Redline R. W. Classification of placental lesions. Am J Obstet Gynecol 213, S21–28, doi:10.1016/j.ajog.2015.05.056 (2015). 26428500

[119] Kim C. J., Romero R., Chaemsaithong P. & Kim J. S. Chronic inflammation of the placenta: definition, classification, pathogenesis, and clinical significance. Am J Obstet Gynecol 213, S53–69, doi:10.1016/j.ajog.2015.08.041 (2015). 26428503 PMC4782598

[120] Romero R. The frequency and type of placental histologic lesions in term pregnancies with normal outcome. J Perinat Med 46, 613–630, doi:10.1515/jpm-2018-0055 (2018). 30044764 PMC6174692

[121] Manichaikul A. Robust relationship inference in genome-wide association studies. Bioinformatics 26, 2867–2873, doi:10.1093/bioinformatics/btq559 (2010). 20926424 PMC3025716

[122] Bray N. L., Pimentel H., Melsted P. & Pachter L. Near-optimal probabilistic RNA-seq quantification. Nat Biotechnol 34, 525–527, doi:10.1038/nbt.3519 (2016). 27043002

[123] Melsted P. Modular and efficient pre-processing of single-cell RNA-seq. bioRxiv, 673285, doi:10.1101/673285 (2019).

[124] Alvarez M. Enhancing droplet-based single-nucleus RNA-seq resolution using the semi-supervised machine learning classifier DIEM. Sci Rep 10, 11019, doi:10.1038/s41598-020-67513-5 (2020). 32620816 PMC7335186

[125] Dobin A. STAR: ultrafast universal RNA-seq aligner. Bioinformatics 29, 15–21, doi:10.1093/bioinformatics/bts635 (2013). 23104886 PMC3530905

[126] Heaton H. Souporcell: robust clustering of single-cell RNA-seq data by genotype without reference genotypes. Nat Methods 17, 615–620, doi:10.1038/s41592-020-0820-1 (2020). 32366989 PMC7617080

[127] Kang H. M. Multiplexed droplet single-cell RNA-sequencing using natural genetic variation. Nat Biotechnol 36, 89–94, doi:10.1038/nbt.4042 (2018). 29227470 PMC5784859

[128] Hafemeister C. & Satija R. Normalization and variance stabilization of single-cell RNA-seq data using regularized negative binomial regression. Genome Biol 20, 296, doi:10.1186/s13059-019-1874-1 (2019). 31870423 PMC6927181

[129] Stuart T. Comprehensive Integration of Single-Cell Data. Cell 177, 1888–1902 e1821, doi:10.1016/j.cell.2019.05.031 (2019). 31178118 PMC6687398

[130] Korsunsky I. Fast, sensitive and accurate integration of single-cell data with Harmony. Nat Methods 16, 1289–1296, doi:10.1038/s41592-019-0619-0 (2019). 31740819 PMC6884693

[131] McInnes L., Healy J. & Melville J. UMAP: Uniform Manifold Approximation and Projection for Dimension Reduction. arXiv:1802.03426 (2018). <https://ui.adsabs.harvard.edu/abs/2018arXiv180203426M≥.

[132] Becht E. Dimensionality reduction for visualizing single-cell data using UMAP. Nat Biotechnol, doi:10.1038/nbt.4314 (2018). 30531897

[133] Aran D. Reference-based analysis of lung single-cell sequencing reveals a transitional profibrotic macrophage. Nat Immunol 20, 163–172, doi:10.1038/s41590-018-0276-y (2019). 30643263 PMC6340744

[134] Love M. I., Huber W. & Anders S. Moderated estimation of fold change and dispersion for RNA-seq data with DESeq2. Genome Biol 15, 550, doi:10.1186/s13059-014-0550-8 (2014). 25516281 PMC4302049

[135] Benjamini Y. & Hochberg Y. Controlling the false discovery rate: a practical and powerful approach to multiple testing. J Roy Statist Soc Ser B 57, 289–300 (1995).

[136] Yu G., Wang L. G., Han Y. & He Q. Y. clusterProfiler: an R package for comparing biological themes among gene clusters. OMICS 16, 284–287, doi:10.1089/omi.2011.0118 (2012). 22455463 PMC3339379

[137] Subramanian A. Gene set enrichment analysis: a knowledge-based approach for interpreting genome-wide expression profiles. Proc Natl Acad Sci U S A 102, 15545–15550, doi:10.1073/pnas.0506580102 (2005). 16199517 PMC1239896

[138] Szklarczyk D. The STRING database in 2017: quality-controlled protein-protein association networks, made broadly accessible. Nucleic Acids Res 45, D362–D368, doi:10.1093/nar/gkw937 (2017). 27924014 PMC5210637

[139] Stano M., Beke G. & Klucar L. viruSITE-integrated database for viral genomics. Database (Oxford) 2016, doi:10.1093/database/baw162 (2016). PMC5199161 28025349

[140] Dickson R. P. Changes in the lung microbiome following lung transplantation include the emergence of two distinct Pseudomonas species with distinct clinical associations. PLoS One 9, e97214, doi:10.1371/journal.pone.0097214 (2014). 24831685 PMC4022512

[141] Kozich J. J., Westcott S. L., Baxter N. T., Highlander S. K. & Schloss P. D. Development of a dual-index sequencing strategy and curation pipeline for analyzing amplicon sequence data on the MiSeq Illumina sequencing platform. Appl Environ Microbiol 79, 5112–5120, doi:10.1128/AEM.01043-13 (2013). 23793624 PMC3753973

[142] Callahan B. J. DADA2: High-resolution sample inference from Illumina amplicon data. Nat Methods 13, 581–583, doi:10.1038/nmeth.3869 (2016). 27214047 PMC4927377

[143] R: A language and environment for statistical computing (R Foundation for Statistical Computing, Vienna, Austria, 2019).

[144] Davis N. M., Proctor D. M., Holmes S. P., Relman D. A. & Callahan B. J. Simple statistical identification and removal of contaminant sequences in marker-gene and metagenomics data. Microbiome 6, 226, doi:10.1186/s40168-018-0605-2 (2018). 30558668 PMC6298009

[145] vegan: Community ecology package v. 2.5–6 (2019).

[146] Anderson M. J. in Wiley StatsRef: Statistics Reference Online 1–15.

