# Supplementary material for: Maternal-Fetal Immune Responses in Pregnant Women Infected with SARS-CoV-2
Source: Res Sq. 2021 Mar 31:rs.3.rs-362886. Preprint. [Version 1] doi: 10.21203/rs.3.rs-362886/v1 (PMC8020997; doi:10.21203/rs.3.rs-362886/v1)
Supplement: Supplement [file feea600990116266462ca122.pdf]

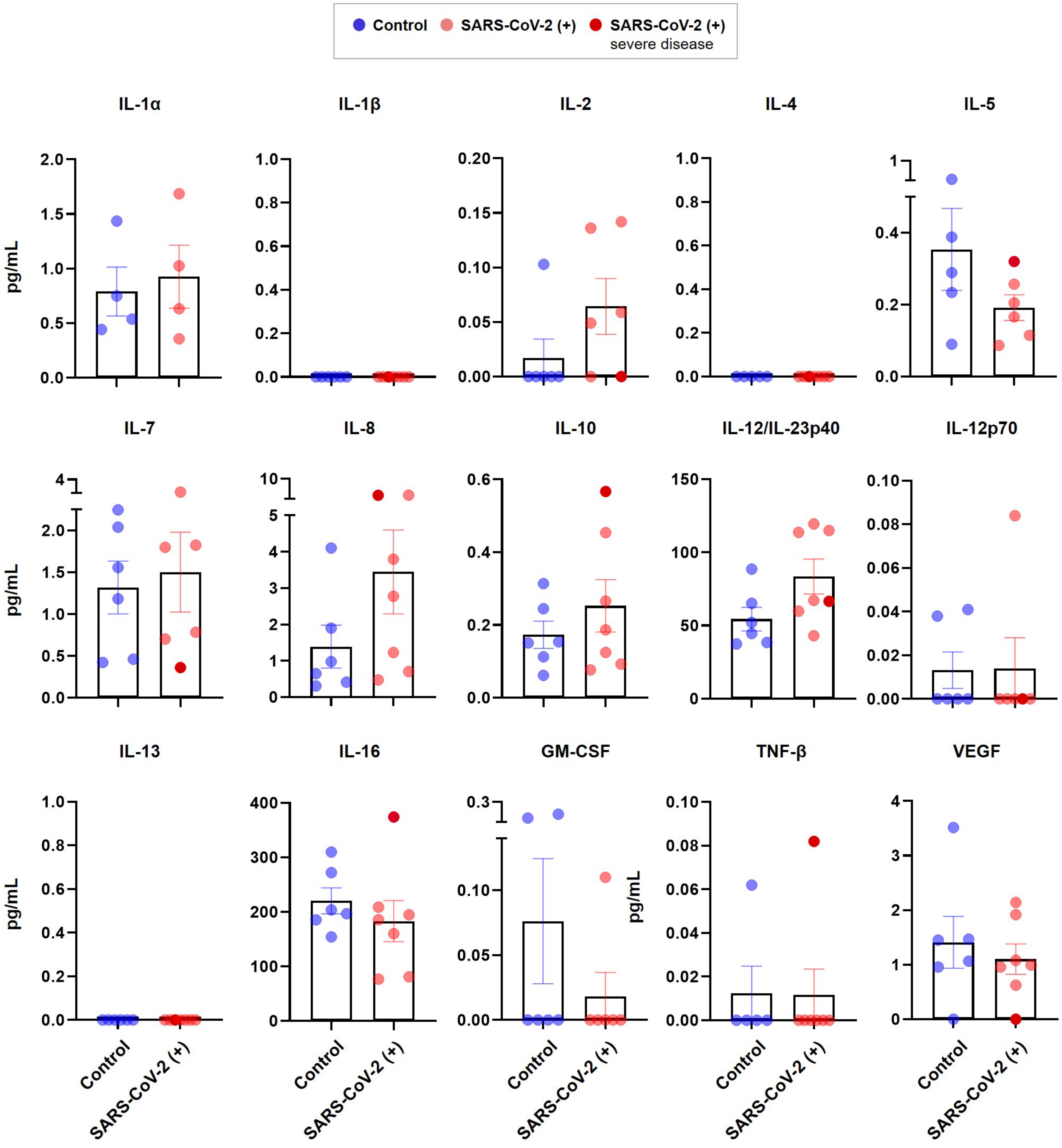

Supplementary Figure 1. Cytokine concentrations in the maternal blood of pregnant women with SARS-CoV-2 infection. Maternal peripheral blood was collected from SARS-CoV-2 (+) and healthy pregnant women upon admission (n = 6-7 per group). Concentrations of IL-1α, IL-1β, IL-2, IL-4, IL-5, IL-7, IL-8, IL-10, IL-12/IL-23p40, IL-12p70, IL-13, IL-16, GM-CSF, TNF-β, and VEGF were measured in the maternal plasma. Bar plots represent mean and standard error of the mean. Differences between groups were evaluated by Mann-Whitney U-tests. P values are considered significant when $p < 0.05$. Blue dots indicate healthy pregnant women, light red dots indicate SARS-CoV-2 (+) pregnant women, and the dark red dot indicates one patient with severe COVID-19 disease.

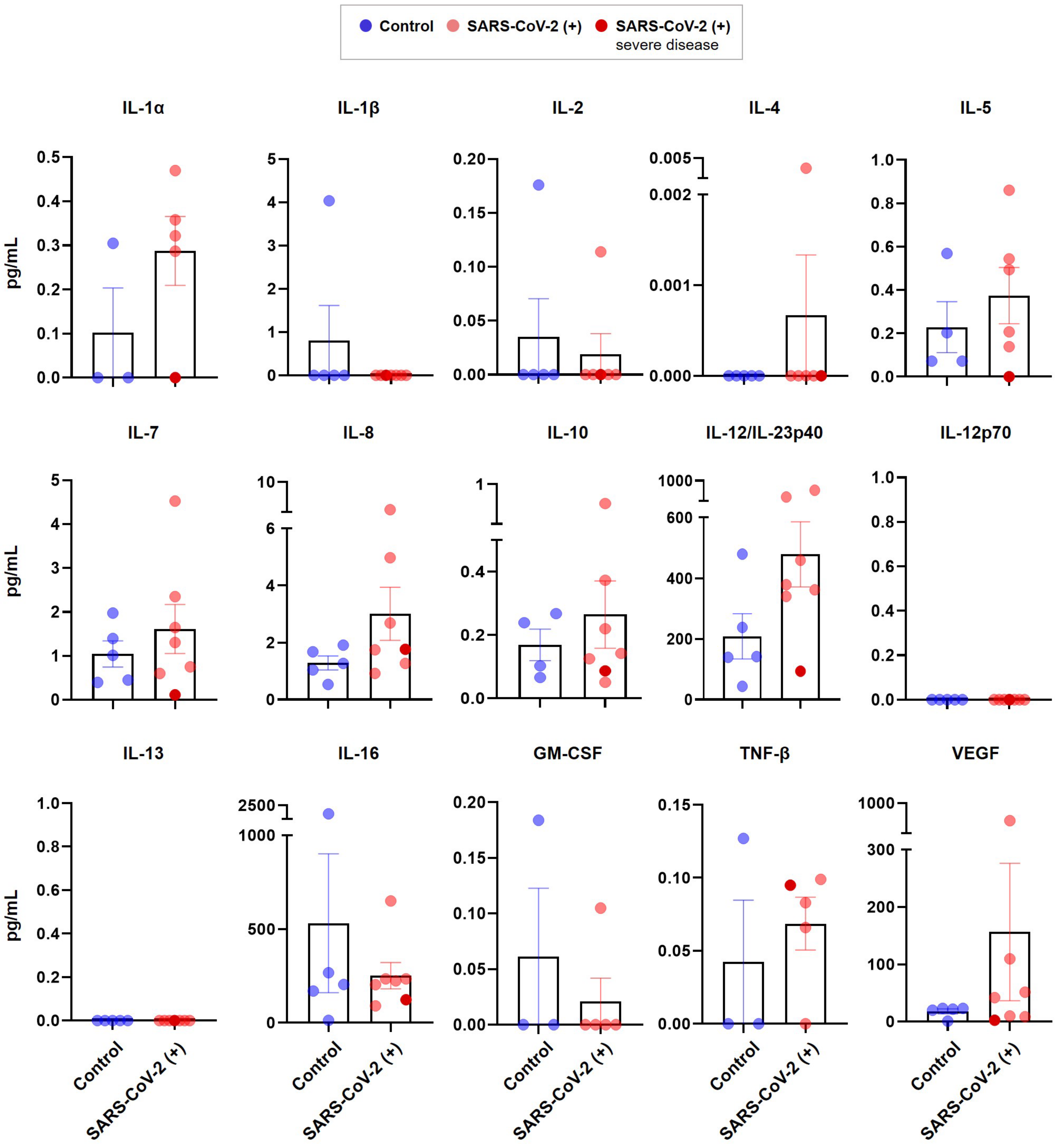

Supplementary Figure 2. Cytokine concentrations in the cord blood of women with SARS-CoV-2 infection. Cord blood was collected from SARS-CoV-2 (+) and healthy women at delivery (n = 5-7 per group). Concentrations of IL-1α, IL-1β, IL-2, IL-4, IL-5, IL-7, IL-8, IL-10, IL-12/IL-23p40, IL-12p70, IL-13, IL-16, GM-CSF, TNF-β, and VEGF were measured in the cord blood plasma. Bar plots represent mean and standard error of the mean. Differences between groups were evaluated by Mann-Whitney U-tests. P values are considered significant when $p < 0.05$. Blue dots indicate healthy pregnant women, light red dots indicate SARS-CoV-2 (+) pregnant women, and the dark red dot indicates one patient with severe COVID-19 disease.

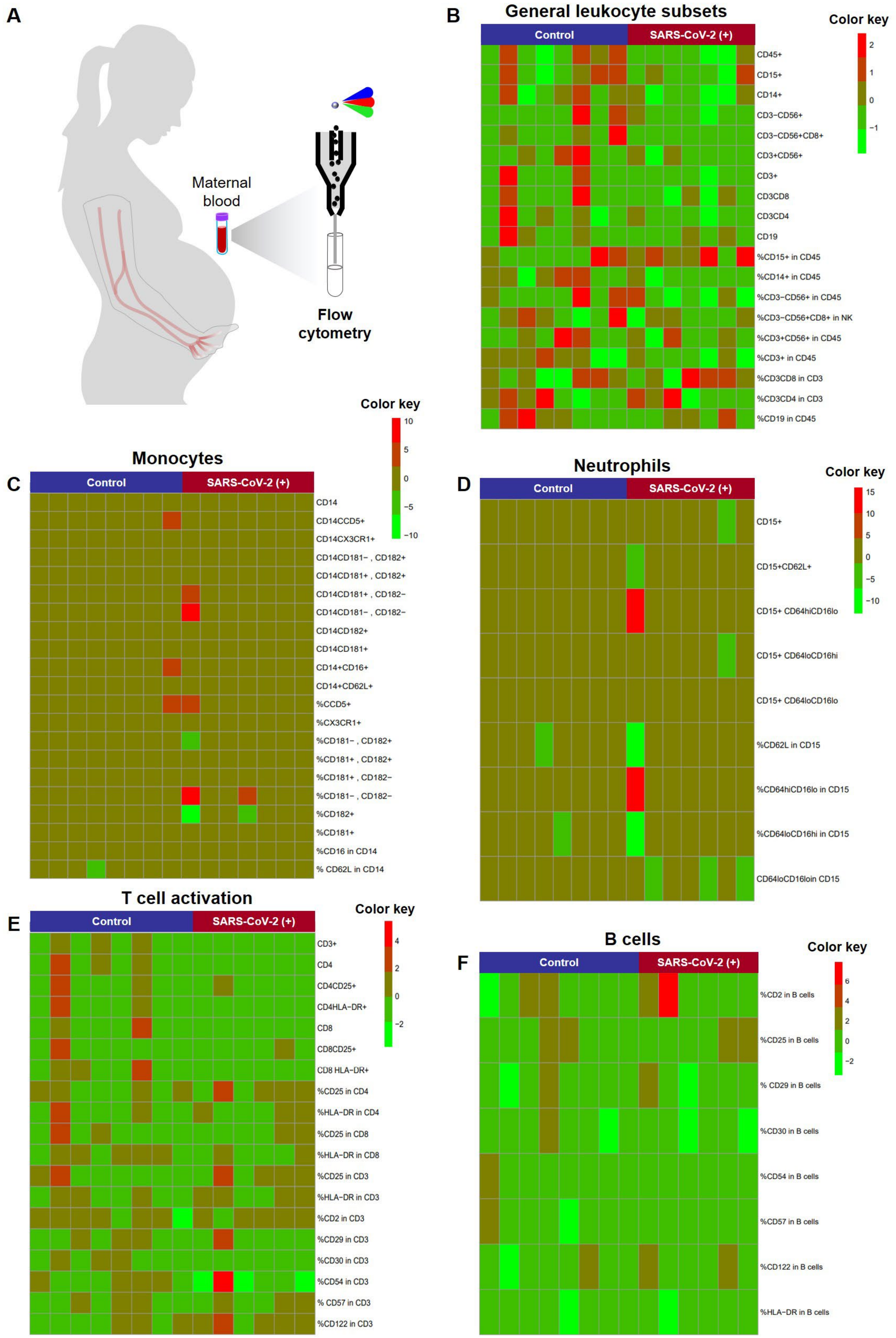

Supplementary Figure 3. Immunophenotyping of leukocyte subsets in the maternal blood of women with SARS-CoV-2 infection. **(A)** Maternal blood was collected from SARS-CoV-2 (+) or healthy pregnant women (n = 7-8 per group) upon admission for immunophenotyping by flow cytometry. Representative heat maps showing the z-scores for **(B)** general leukocyte subpopulations, **(C)** monocyte subsets, **(D)** neutrophil subsets, **(E)** activated T cell subsets, and **(F)** B cell subsets. Abundances are calculated using z-scores from the maternal blood of SARS-CoV-2 (+) and healthy pregnant patients, where red and green indicate increased and decreased abundance, respectively. Cell numbers and/or proportions are shown.

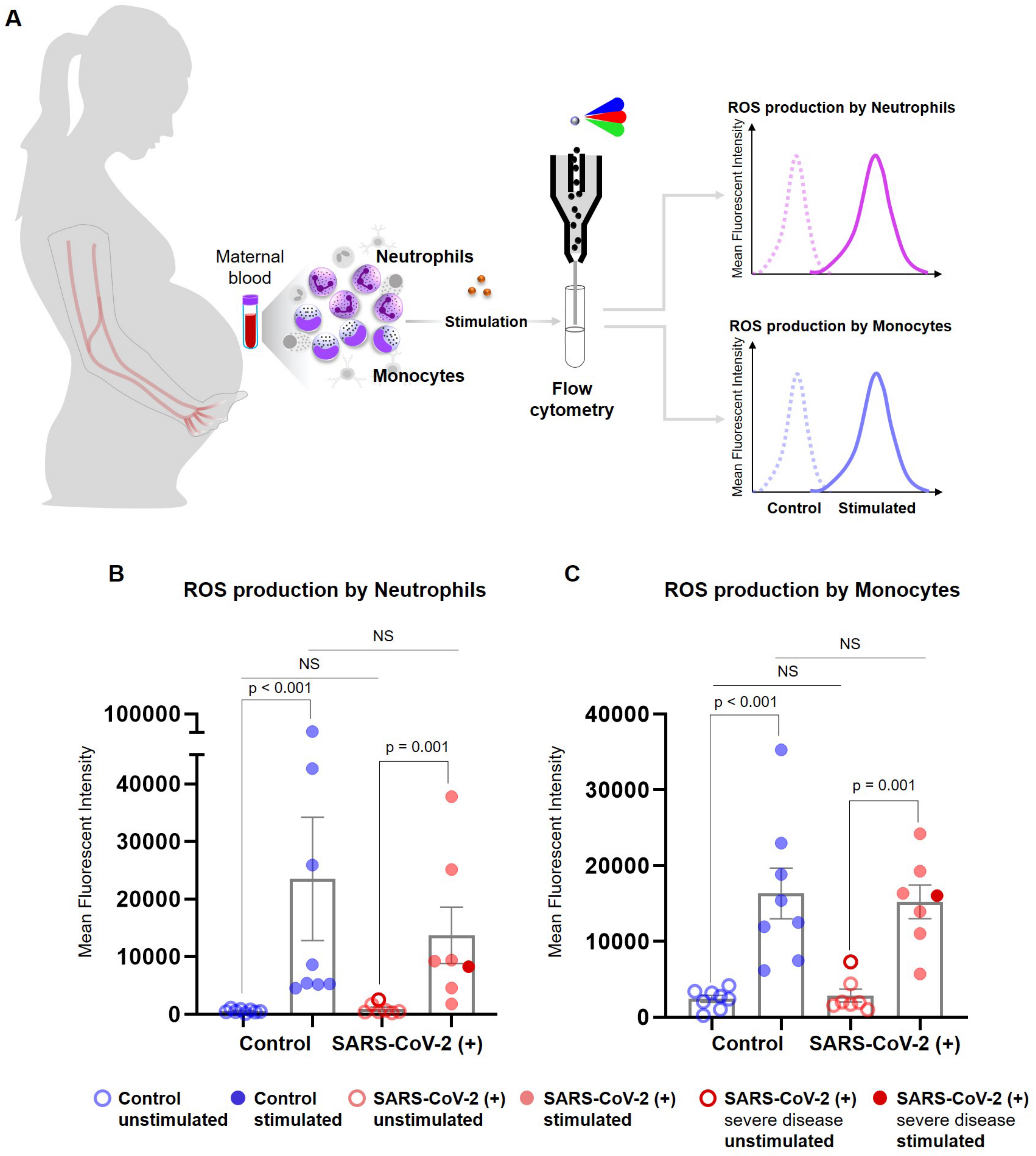

Supplementary Figure 4. Reactive oxygen species production by peripheral neutrophils and monocytes in pregnant women with SARS-CoV-2 infection. (A) Maternal blood was collected from SARS-CoV-2 (+) or healthy pregnant women (n = 7-8 per group) upon admission, and leukocytes were stimulated in vitro prior to the evaluation of reactive oxygen species (ROS) production by neutrophils and monocytes using flow cytometry. **(B)** Bar plots showing ROS production by neutrophils in SARS-CoV-2 (+) or healthy pregnant women with or without stimulation as represented by mean fluorescence intensity. **(C)** Bar plots showing ROS production by monocytes in SARS-CoV-2 (+) or healthy pregnant women with or without stimulation. Data are represented as mean and standard error of the mean. Differences between groups were evaluated by Mann-Whitney U-tests. P values are considered significant when $p < 0.05$. Open dots indicate unstimulated cells and filled dots indicate stimulated cells. Blue dots indicate healthy pregnant women, light red dots indicate SARS-CoV-2 (+) pregnant women, and the dark red dot indicates one patient with severe COVID-19 disease.

A

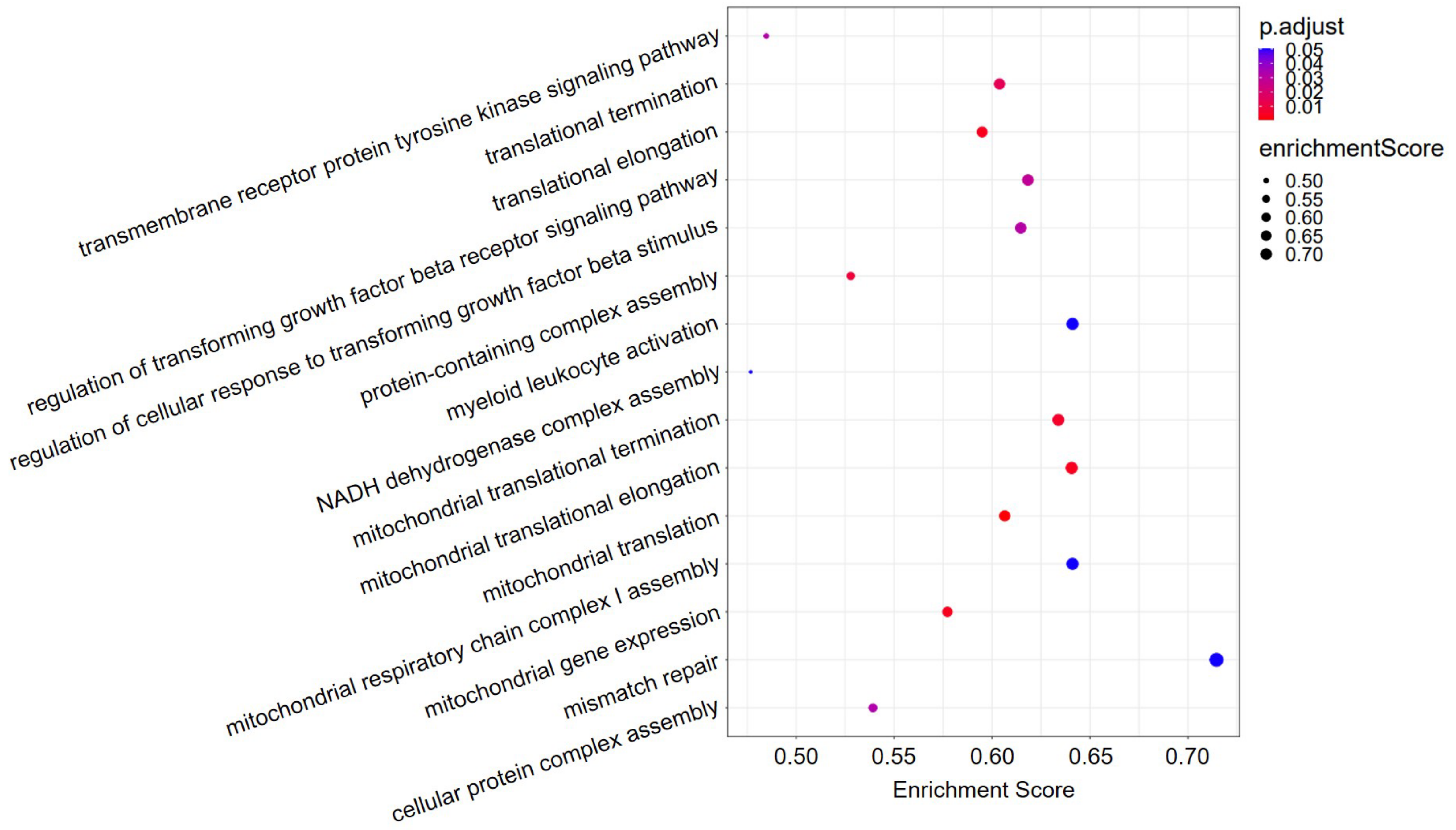

B

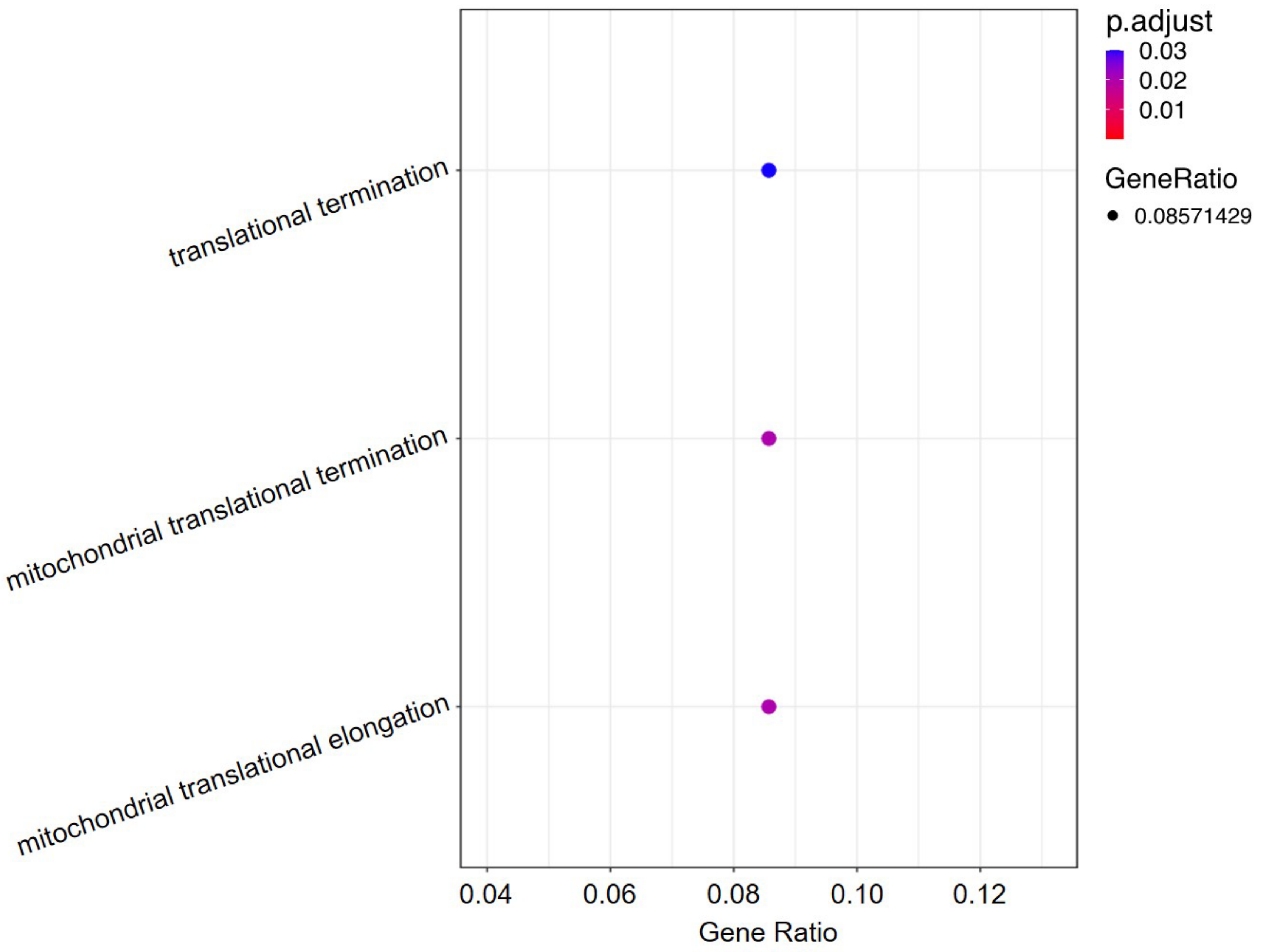

Supplementary Figure 5. Gene ontology (GO) terms shared between maternal T cells from the chorioamniotic membranes of SARS-CoV-2 (+) pregnant women and maternal peripheral T cell signatures from a previous report (Meckiff et al., 2020). (A) Over-representation analysis reporting enriched GO terms that are significant with $q < 0.05$. **(B)** Gene set enrichment analysis reporting enriched GO terms that are significant with $q < 0.05$.

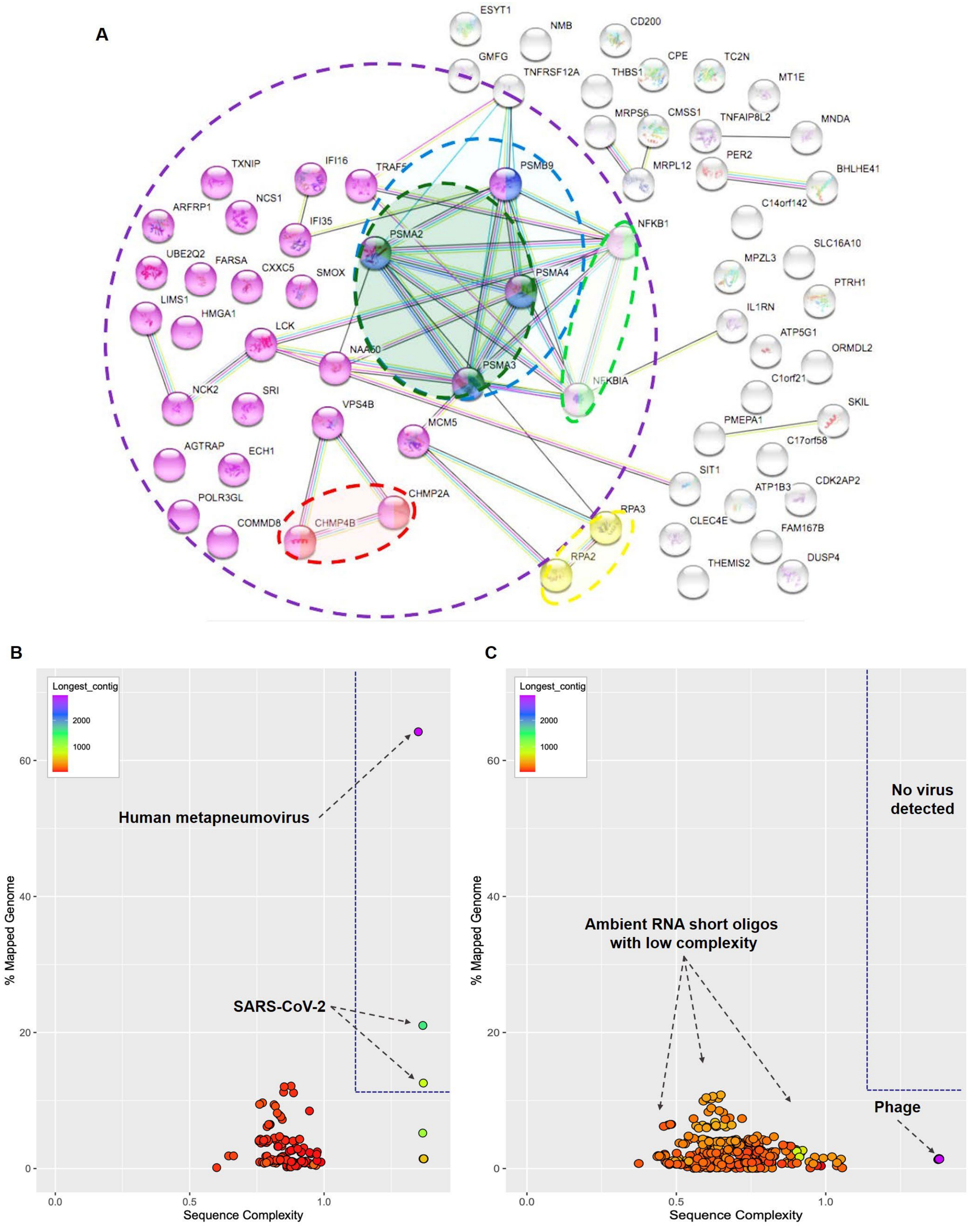

Supplementary Figure 6. STRING enrichment analysis and viral detection in pregnant women SARS-CoV-2 infection. (A) The STRING database was used to explore significant gene ontology (GO) terms obtained from the single-cell RNA-seq data from the chorioamniotic membranes (CAM) and placental villi and basal plate (PVBP) of SARS-CoV-2 (+) or healthy pregnant women (n = 7-8 per group). Genes are circled based on their associated GO terms: cytosol (purple), DNA replication factor A complex (yellow), ESCRT III complex (red), I-kappa B /NF-kappaB complex (light green), proteasome core complex (blue), and proteasome core complex, alpha-subunit complex (dark green). Viral-Track scatter plots showing the presence of viral sequences in single-cell RNA-seq reads from **(B)** bronchoalveolar lavage of SARS-CoV-2 (+) patients, previously reported data (Bost et al., 2020), and **(C)** single-cell RNA-seq data from the CAM and PVBP of SARS-CoV-2 (+) and pregnant healthy women. Blue dashed lines represent thresholds for viral detection, with the virus name indicated by arrows.

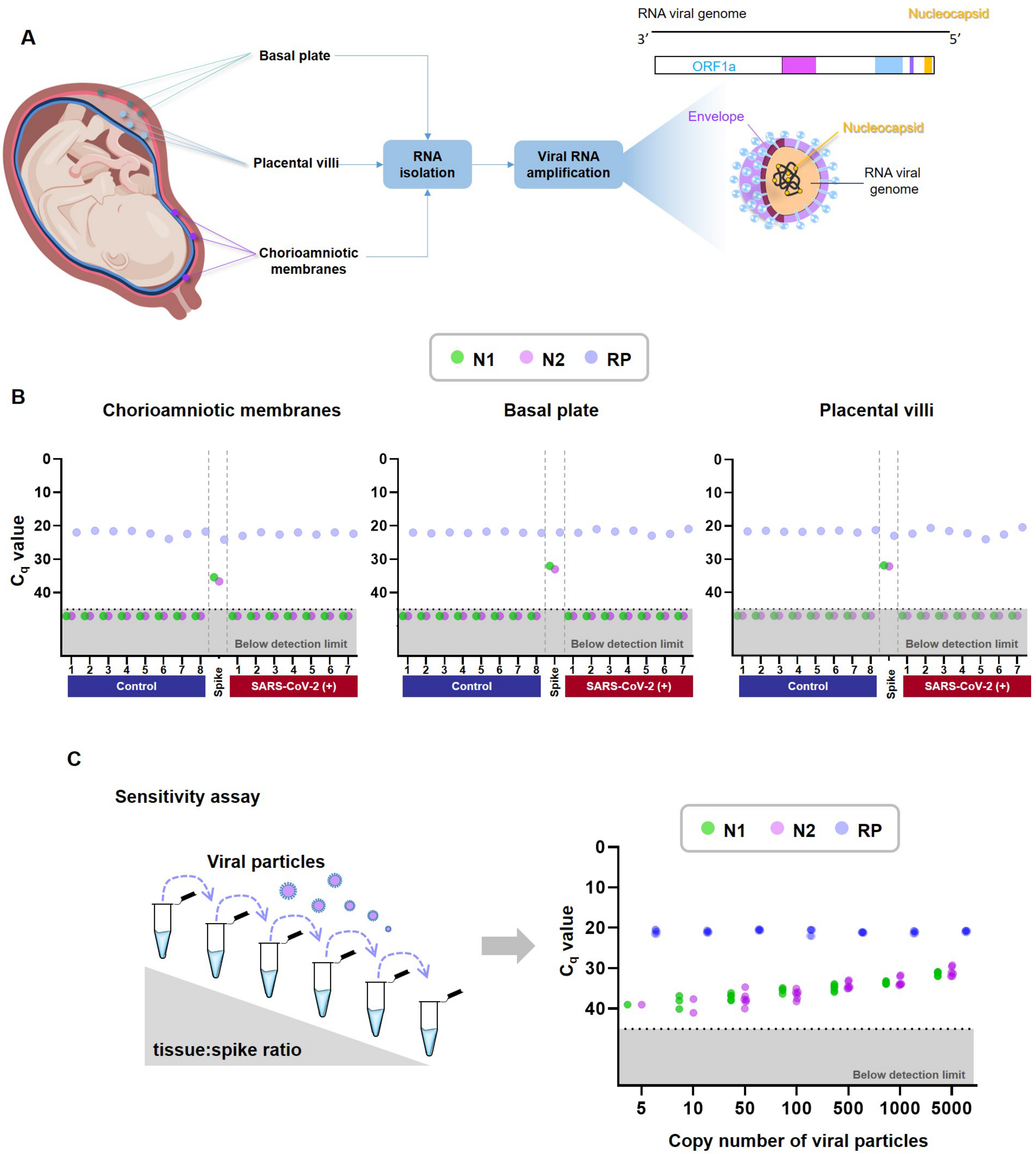

Supplementary Figure 7. Molecular detection of SARS-CoV-2 viral RNA in the placenta and maternal-fetal interface of women with SARS-CoV-2 infection. (A) Schematic representation showing the isolation of viral RNA from the chorioamniotic membranes, basal plate, and placental villi of SARS-CoV-2 (+) and healthy pregnant women (n = 7-8 per group). **(B)** RT-qPCR results of SARS-CoV-2 viral RNA detection in the chorioamniotic membranes, basal plate, and placental villi. **(C)** Sensitivity assay performed to measure the lowest detectable amount of viral particles from SARS-CoV-2. N1 and N2 denote two SARS-CoV-2 nucleocapsid (N) genes, and RP denotes the RNase P gene that serves as positive internal PCR control. Undetermined quantification cycle (Cq) values are represented below the detection limit (gray area).

Supplementary Table 1. Clinical and demographic characteristics of pregnant women with SARS-CoV-2 infection and healthy pregnant women.

	**Controls (n = 8)**	**SARS-CoV-2 (+) (n = 7)**	**p-value**
Maternal age (years; median [IQR])[a]	30 (27-31.3)	26 (21-29)	0.27
Body mass index (kg/m^2; median [IQR])[a]	34.4 (32.6-39.3)	29.2 (28.1-34.4)	0.15
Primiparity[b]	12.5% (1/8)	14.3 % (1/7)	1.0
Race/ethnicity[b]			1.0
African-American	100% (8/8)	100% (7/7)	
White	0% (0/8)	0% (0/7)	
Other	0% (0/8)	0% (0/7)	
Symptoms			-
None	-	71.4% (5/7)	
Mild	-	14.3% (1/7)	
Severe	-	14.3% (1/7)	
Gestational age at delivery (weeks; median [IQR])[a]	38.6 (38.4-39.3)	38.6 (38.4-38.9)	0.85
Labor at admission[b]	75% (6/8)	71.4% (5/7)	1.0
Cesarean section[b]	37.5% (3/8)	28.6% (2/7)	1.0
Preeclampsia[b]	0% (0/8)	14.3% (1/7)	0.47
Birthweight (grams; median [IQR])[a]	3177.5 (2805-3553.8)	3210 (2902.5-3230)	0.61
Apgar score at 1 min (median [IQR])[a]	8 (8-8)	8 (8-8.5)	0.12
Apgar score at 5 min (median [IQR])[a]	9 (9-9)	9 (9-9)	1.0
Acute maternal inflammatory response[b]			
Stage 1 (Early acute subchorionitis or chorionitis)	12.5% (1/8)	0% (0/7)	1.0
Stage 2 (Acute chorioamnionitis)	0% (0/8)	14.3% (1/7)	0.47
Stage 3 (Necrotizing chorioamnionitis)	0% (0/8)	0% (0/7)	1.0
Acute fetal inflammatory response[b]			
Stage 1 (Chorionic vasculitis or umbilical phlebitis)	0% (0/8)	14.3% (1/7)	0.47
Stage 2 (Umbilical arteritis)	0% (0/8)	0% (0/7)	1.0
Stage 3 (Necrotizing funisitis)	0% (0/8)	0% (0/7)	1.0
Chronic maternal inflammatory response[b]			
Chronic deciduitis	0% (0/8)	0% (0/7)	1.0
Chronic villitis of unknown etiology	25% (2/8)	0% (0/7)	0.47
Chronic chorioamnionitis	0% (0/8)	0% (0/7)	1.0
Chronic fetal inflammatory response[b]			
Eosinophilic T-cell vasculitis	0% (0/8)	14.3% (1/7)	0.47
Other placental lesions			
Maternal vascular malperfusion[b]	12.5% (1/8)	0% (0/7)	1.0
Fetal vascular malperfusion[b]	0% (0/8)	0% (0/7)	1.0
Perivillous fibrin deposition[b]	25% (2/8)	28.6% (2/7)	1.0
Intervillous thrombus[b]	12.5% (1/8)	28.6% (2/7)	0.57
Intraplacental thrombus[b]	12.5% (1/8)	14.3% (1/7)	1.0

Data are given as median (interquartile range, IQR) and percentage (n/N).
P values < 0.05 are considered significant
[a]Mann-Whitney U test.
[b]Fisher's exact test.

Supplementary Table 2. Log2 fold change in maternal and neonatal cytokine responses of SARS-CoV2 (+) pregnant women.

Cytokines	log2FC	p-value	Specimen
**IFN-γ**	1.84	0.171	Maternal Blood
**IL-10**	0.37	0.628	Maternal Blood
**IL-12/IL-23p40**	0.60	0.073	Maternal Blood
**IL-15**	0.43	0.022	Maternal Blood
**IL-16**	-0.41	0.445	Maternal Blood
**IL-17A**	0.65	0.662	Maternal Blood
**IL-1α**	0.15	0.886	Maternal Blood
**IL-5**	-0.70	0.247	Maternal Blood
**IL-6**	-0.66	0.432	Maternal Blood
**IL-7**	0.08	1.000	Maternal Blood
**IL-8**	1.25	0.181	Maternal Blood
**TNF**	0.28	0.836	Maternal Blood
**VEGF**	-0.35	0.662	Maternal Blood
**IFN-γ**	-0.50	0.267	Cord Blood
**IL-10**	0.30	0.927	Cord Blood
**IL-12/IL-23p40**	1.32	0.149	Cord Blood
**IL-15**	0.03	0.639	Cord Blood
**IL-16**	0.15	1.000	Cord Blood
**IL-17A**	1.61	0.036	Cord Blood
**IL-5**	1.23	0.268	Cord Blood
**IL-6**	-2.90	0.017	Cord Blood
**IL-7**	0.24	0.639	Cord Blood
**IL-8**	0.99	0.149	Cord Blood
**TNF**	1.01	0.048	Cord Blood
**VEGF**	1.56	0.432	Cord Blood

*$p < 0.05$, considered significant, by Wilcoxon Test

Supplementary Table 4. Summary of slides used for immunohistological detection of SARS-CoV-2 spike and nucleocapsid proteins. Number of slides used for mmunohistochemistry staining at each placental location (placental villi, basal plate, and chorioamniotic membranes) from SARS-CoV-2 (+) and healthy pregnant women. Internal controls used include spiked or non-spiked tissues from healthy pregnant women.

Group	Individual	Number of slides processed for immunological detection of SARS-CoV-2 proteins			
		Placenta villi	Basal plate	Chorioamniotic membranes	Total
Internal controls	Non-spiked tissue from healthy pregnant woman	2	2	2	6
	Spiked tissue from healthy pregnant woman	2	2	2	6
Healthy pregnant women	#1	14	10	6	30
	#2	14	10	6	30
	#3	14	10	6	30
SARS-CoV-2 (+) pregnant women	#1 (severe COVID-19 disease)	14	10	6	30
	#2	14	10	6	30
	#3	14	10	6	30
	#4	14	10	6	30
	#5	14	10	6	30
	#6	14	10	6	30
	#7	14	10	6	30
Total number of slides		144	104	64	312

Supplementary Table 5. Bacterial load in the placenta of women with SARS-CoV-2 infection. Comparison of bacterial load in the chorioamniotic membranes, placental amnion-chorion, and placental villous tree from SARS-CoV-2 (+) pregnant women who delivered by cesarean section or vaginally (n = 7) and from healthy pregnant women who delivered by cesarean section or vaginally (n = 8). The table shows no difference in the likelihood of having a bacterial load exceeding that of technical controls for background DNA contamination (i.e. blank DNA extraction kits).

	**Control**	**COVID-19**	**Proportion z-test**
**Cesarean delivery**			
Chorioamniotic membranes	1/3 (33%)	0/2 (0%)	z = 0.907, p = 0.545
Placental amnion-chorion	0/3 (0%)	1/2 (50%)	z = -1.369, p = 0.513
Placental villous tree	0/3 (0%)	2/2 (100%)	z = -2.236, p = 0.151
**Vaginal delivery**			
Chorioamniotic membranes	5/5 (100%)	5/5 (100%)	z = 0.000, p = 1.000
Placental amnion-chorion	5/5 (100%)	5/5 (100%)	z = 0.000, p = 1.000
Placental villous tree	4/5 (80%)	5/5 (100%)	z = -1.054, p = 0.545

[a] Two-tailed, with Benjamini-Hochberg corrections applied

Supplementary Table 6. Antibodies used for immunophenotyping

Antibody	Fluorophore	Clone	Company
Isotype	PE	G155-178	BD Biosciences
Isotype	PerCPCy5.5	MOPC-21	BD Biosciences
Isotype	FITC	X40	BD Biosciences
Isotype	A647	MOPC-21	BioLegend
Isotype	BV421	27-35	BD Biosciences
Isotype	BV605	MOPC-21	BioLegend
Isotype	Alexa594	R3-34	BD Biosciences
Isotype	PE-Cy7	RTK4530	BioLegend
Isotype	BV711	X40	BD Biosciences
Isotype	PE-Cy5	Clone MOPC-21	BD Biosciences
Isotype	APC	Clone MOPC-21	BD Biosciences
Isotype	PE	Clone MOPC-21	BD Biosciences
Isotype	PE-CF594	Clone X40	BD Biosciences
Isotype	BV650	Clone X40	BD Biosciences
Isotype	BV786	Clone R3-34	BD Biosciences
Isotype	BV510	Clone R35-95	BD Biosciences
Isotype	BV711	Clone R3-34	BD Biosciences
Isotype	Alexa488	Clone 27-35	BD Biosciences
Isotype	Alexa700	MOPC-21	BD Biosciences
Isotype	PE	R3-34	BD Biosciences
Isotype	Alexa647	MOPC-21	BD Biosciences
Universal mouse isotype	-	-	Agilent
Universal rabbit isotype	-	-	Agilent
CD3	BUV737	UCHT1 (also known as UCHT-1, UCHT 1)	BD Biosciences
CD4	APC-H7	RPA-T4	BD Biosciences
CD8a	BUV395	RPA-T8	BD Biosciences
CD45RA	Alexa 700	HI100	BD Biosciences
CD196 (CXCR6)	Alexa 488	G034E3	BioLegend
CD197 (CCR7)	PE-Cy7	3D12	BD Biosciences
CD45	V450	HI30	BD Biosciences
CD183 (CXCR3)	APC	1C6/CXCR3 (also known as 1C6, LS177-1C6)	BD Biosciences
CD3	APC-H7	SK7	BD Biosciences
CD19	PE-Cy5	HIB19	BD Biosciences
CD14	BUV395	MφP9	BD Biosciences
CD45	Alexa700	HI30	BD Biosciences
CD4	BUV737	SK3	BD Biosciences
CD15	BV650	HI98	BD Biosciences
CD8	BV786	RPA-T8	BD Biosciences
CD56	BV711	NCAM16.2	BD Biosciences
MIP1α	PE	11A3	BD Biosciences
MIP1β	PerCPCy5.5	D21-1351	BD Biosciences
IL-1β	FITC	AS5	BD Biosciences
IL-1β	A647	JK1B-1	BioLegend

IL-8	BV421	G265-8	BD Biosciences
TNFα	BV605	MAb11	BioLegend
IL-6	Alexa594	MQ2-13A5	BD Biosciences
CX3CR1	PE-Cy7	2A9-1	BioLegend
CCR5 (CD195)	BV711	2D7/CCR5	BD Biosciences
CD181 (CXCR1)	PE-Cy5	5A12	BD Biosciences
CD182 (CXCR2)	APC	6C6	BD Biosciences
IL-1β	FITC	AS10	BD Biosciences
IL-1RA	PE	AS17	BD Biosciences
CD103	PE-Cy7	Ber-ACT8	BioLegend
Granzyme B	PE-CF594	GB11	BD Biosciences
IFNɣ	BV650	4S.B3	BD Biosciences
IL-10	BV786	JES3-9D7	BD Biosciences
IL-2	BV510	MQ1-17H12	BD Biosciences
IL-4	BV711	MP4-25D2	BD Biosciences
Perforin	Alexa488	δG9	BD Biosciences
IL-17A	Alexa700	N49-653	BD Biosciences
IL-5	PE	TRFK5	BD Biosciences
IL-9	PerCP-Cy5.5	MH9A3	BD Biosciences
T-bet	Alexa647	4B10	BD Biosciences
CD62L	BV650	DREG-56	BD Biosciences
CD45RO	PE-Cy5	UCHL1	BD Biosciences
CD300a	PE	E59.126	Beckman Coulter
HLA-DR	PE-CF594	G46-6	BD Biosciences
CD2	BV421	RPA-2.10	BD Biosciences
CD19	Alexa488	HIB19	BD Biosciences
CD25	PE-Cy7	M-A251	BD Biosciences
CD29	BV510	MAR4	BD Biosciences
CD30	APC	BerH8	BD Biosciences
CD54	BV711	HA58	BD Biosciences
CD57	BV605	QA17A04	BioLegend
CD122 (IL-2Rβ)	PE	Clone TU27	BioLegend
CD10	BUV395	HI10a	BD Biosciences
CD13	PE-Cy7	L138	BD Biosciences
CD14	BV650	M5E2	BD Biosciences
CD15	BV605	W6D3	BD Biosciences
CD16	PerCP-Cy5.5	Clone 3G8	BD Biosciences
CD33	BV711	WM53	BD Biosciences
CD62L	PE-CF594	DREG-56	BD Biosciences
CD123	BV786	7G3	BD Biosciences
CD185	Alexa700/APC-Cy5.5	J252D4	BioLegend
CD203c	BV421	NP4D6	BD Biosciences
CD279	FITC	MIH4	BD Biosciences
CD64	APC-H7	10.1	BD Biosciences
CD303	APC/Alexa647	201A	BioLegend
CD83	PE-Cy5	HB15e	BD Biosciences
CD172b	PE	B4B6	BD Biosciences
